# Enhancing autophagy maturation with CCZ1-MON1A complex alleviates neuropathology and memory defects in Alzheimer disease models

**DOI:** 10.7150/thno.64148

**Published:** 2022-01-24

**Authors:** Cui-Zan Cai, Xu-Xu Zhuang, Qi Zhu, Ming-Yue Wu, Huanxing Su, Xiao-jin Wang, Ashok Iyaswamy, Zhenyu Yue, Qian Wang, Bin Zhang, Yu Xue, Jieqiong Tan, Min Li, Huanhuan He, Jia-Hong Lu

**Affiliations:** 1 State Key Laboratory of Quality Research in Chinese Medicine, Institute of Chinese Medical Sciences, University of Macau, Macau SAR, China; 2 Molecular Imaging Center, Guangdong Provincial Key Laboratory of Biomedical Imaging, The Fifth Affiliated Hospital, Sun Yat-sen University, Zhuhai, 519000, China; 3 Mr. and Mrs. Ko Chi Ming Centre for Parkinson's Disease Research, School of Chinese Medicine, Hong Kong Baptist University, Hong Kong SAR, China; 4 Department of Neurology, The Friedman Brain Institute, Icahn School of Medicine at Mount Sinai, New York, NY, 10029, USA; 5 Department of Genetics and Genomic Sciences, Icahn School of Medicine at Mount Sinai, New York, NY, 10029, USA; 6 Mount Sinai Center for Transformative Disease Modeling, Icahn School of Medicine at Mount Sinai, New York, NY 10029, USA; 7 Department of Pharmacological Sciences, Icahn School of Medicine at Mount Sinai, New York, NY 10029, USA; 8 Icahn Genomics Institute, Icahn School of Medicine at Mount Sinai, New York, NY 10029, USA; 9 Key Laboratory of Molecular Biophysics of Ministry of Education, Hubei Bioinformatics and Molecular Imaging Key Laboratory, Center for Artificial Intelligence Biology, College of Life Science and Technology, Huazhong University of Science and Technology, Wuhan, China; 10 Center for Medical Genetics, School of Life Sciences, Central South University, Changsha 410078, China

**Keywords:** Alzheimer's disease, Aβ, Autophagy, CCZ1-MON1A, RAB7

## Abstract

**Rationale:** Impairment of autophagy maturation has been implicated in Alzheimer's disease (AD) pathogenesis. However, the mechanism for this impairment has not been elucidated, and whether enhancing autophagy maturation is a viable therapeutic strategy for AD has not been verified.

**Methods:** We examined the autophagosome maturation process in AD cell and mouse models by immunoblotting. To further understand the changes in autophagy in AD brains, we analyzed the transcriptome by RNA-sequencing and measured the expression of RAB7, CCZ1 and MON1A.

We performed brain stereotaxic injections of AAV into 3xTg AD mouse brain and WT mouse brain to over-express MON1A/CCZ1 or knockdown MON1A. For in vitro studies, we purified autophagosomes, and determined GTP-RAB7 level in autophagosome fractions by GST-R7BD affinity-isolation assay.

**Results:** We report that the active form of RAB7 was selectively decreased in autophagosome fractions isolated from cells and tissues of AD models, and that this decrease was accompanied by impaired activity of its guanine nucleotide exchange factor (GFE) CCZ1-MON1A. Overexpressing CCZ1-MON1A increased the active form of RAB7, enhanced autophagosome maturation, and promoted degradation of APP-CTFs, Aβ and P-tau in an autophagy-dependent manner in cells and a mouse AD model.

**Conclusions:** Our data reveals that CCZ1-MON1A-RAB7 complex dysfunction is a potential mechanism for autophagosome maturation defects in AD, and advances the possibility that enhancing autophagosome maturation is a novel therapeutic strategy against AD.

## Introduction

Alzheimer's disease (AD) is the most common neurodegenerative disorder worldwide. It is characterized by two main histological hallmarks: amyloid plaques composed of Aβ peptides, and neurofibrillary tangles (NFTs) formed by hyperphosphorylated tau protein 1, 2. Aβ is generated through cleavage of APP by β- and γ-secretases. APP is first cleaved by BACE1/β-secretase, which generates soluble sAPPβ and membrane-bound C99 APP-CTFβ. Subsequently APP-CTFβ is cleaved into Aβ1-40 or Aβ1-42 by γ-secretase 3. Tremendous efforts have been spent on the development of therapies targeting Aβ and P-tau for AD treatment; however, these attempts have largely failed. There is a huge need for the discovery of novel therapeutic strategy(s) to slow disease progression. Macroautophagy (henceforth referred to as autophagy) is an evolutionarily conserved pathway that regulates the degradation of cytoplasm materials including long-lived proteins, damaged cellular organelles and aggregated or misfolded proteins 4. Autophagy features the formation of double-membrane autophagosomes that fuse with lysosomes for degradation. It can be divided into three stages: initiation, extension, and maturation. Massive accumulation of abnormal autophagosomes in neurons has been observed in the brains of AD patients 5, implicating impairment of autophagy, especially at the autophagosome maturation stage, in the pathogenesis of AD 6. Furthermore, increasing evidence has revealed that disruption of the autophagy-lysosome pathway occurs very early in disease progression 7. Inger Lauritzen demonstrated a link between accumulating C99 and the endosomal-autophagic-lysosomal pathology. They found that C99-mediated autophagy impairment is independent of Aβ 8. Similarly, Loan Vaillant-Beuchot found that APP-CTF accumulation also impaired autophagy/mitophagy as shown by the accumulation of LC3-II and SQSTM1/p62, impaired Parkin and PINK1 recruitment to mitochondria, up-regulation of membrane and matrix mitochondrial proteins, and defective fusion of mitochondria with lysosomes 9. However, the mechanisms underlying autophagy impairment in AD are not fully understood.

Modulation of the autophagy pathway has been recognized as a potential therapeutic strategy for treating neurodegenerative diseases including AD. Many studies have been performed to test whether enhancement of autophagy by genetic or pharmacological approaches can lead to improvement of AD phenotypes in AD models 10-12. However, autophagy is a double-edged sword in AD development, in that simply enhancing autophagosome induction without restoring the autophagosome maturation defect may have detrimental consequences for AD 13. A wiser strategy for modulating autophagy in AD may be specifically targeting the autophagosome maturation process. However, the feasibility and efficacy of this strategy has not yet been verified. RAB7 is a small GTPase required for both endosome and autophagosome maturation 14. CCZ1-MON1A complex is the RAB7 guanine nucleotide exchange factor (GEF) which promotes the exchange of GDP-RAB7 (inactive state) to GTP-RAB7 (active state) 15, 16. Krisztina Hegedus found that, in starved drosophila fat cells, autophagosomes accumulate due to impaired fusion with lysosomes upon loss of the CCZ1-MON1A-RAB7 module. They also found that CCZ1-MON1A recruits RAB7 to autophagosomes and regulates autophagosome-lysosome fusion in these cells 17. The yeast homolog of the members of the mammalian LC3 protein (Atg8) directly binds to CCZ1 and is indeed a primary determinant to recruitment of CCZ1-MON1A to autophagosomes 18. Our previous research showed that over-expression of the constantly active form of RAB7 rescued autophagosome maturation defects in a cellular AD model lacking autophagy gene *NRBF2*. Meanwhile, our data revealed that APP interacts and co-localizes with CCZ1 and MON1A in N2S cells 19. These data highlight the role of CCZ1-MON1A-RAB7 in regulating autophagosome maturation for efficient degradation of AD-associated proteins. We hence propose that the CCZ1-MON1A complex is a key determinant of autophagosome maturation in AD and that enhancing this complex's activity could be an effective approach to facilitating autophagosome maturation in AD models for the alleviation of AD pathologies.

In this paper, we report the reduction of the active form RAB7 in the autophagosome fraction isolated from cellular and mice AD models, as well as the inhibition of CCZ1-MON1A GEF activity in cellular and mice AD models. Our results verify the effects of CCZ1-MON1A in regulating autophagosome maturation, in degrading AD-related protein, and in improving neuropathological and behavioral performance in AD models. Our results suggest that CCZ1-MON1A, via activating autophagosome maturation, facilitates AD-related protein degradation and alleviates memory defects and neuropathological abnormalities in AD. Our findings provide evidence that enhancement of autophagosome maturation is of potential therapeutic value in AD treatment.

## Results

### AD cell and mice models exhibit inhibition of autophagosome maturation

**Figure 1:**
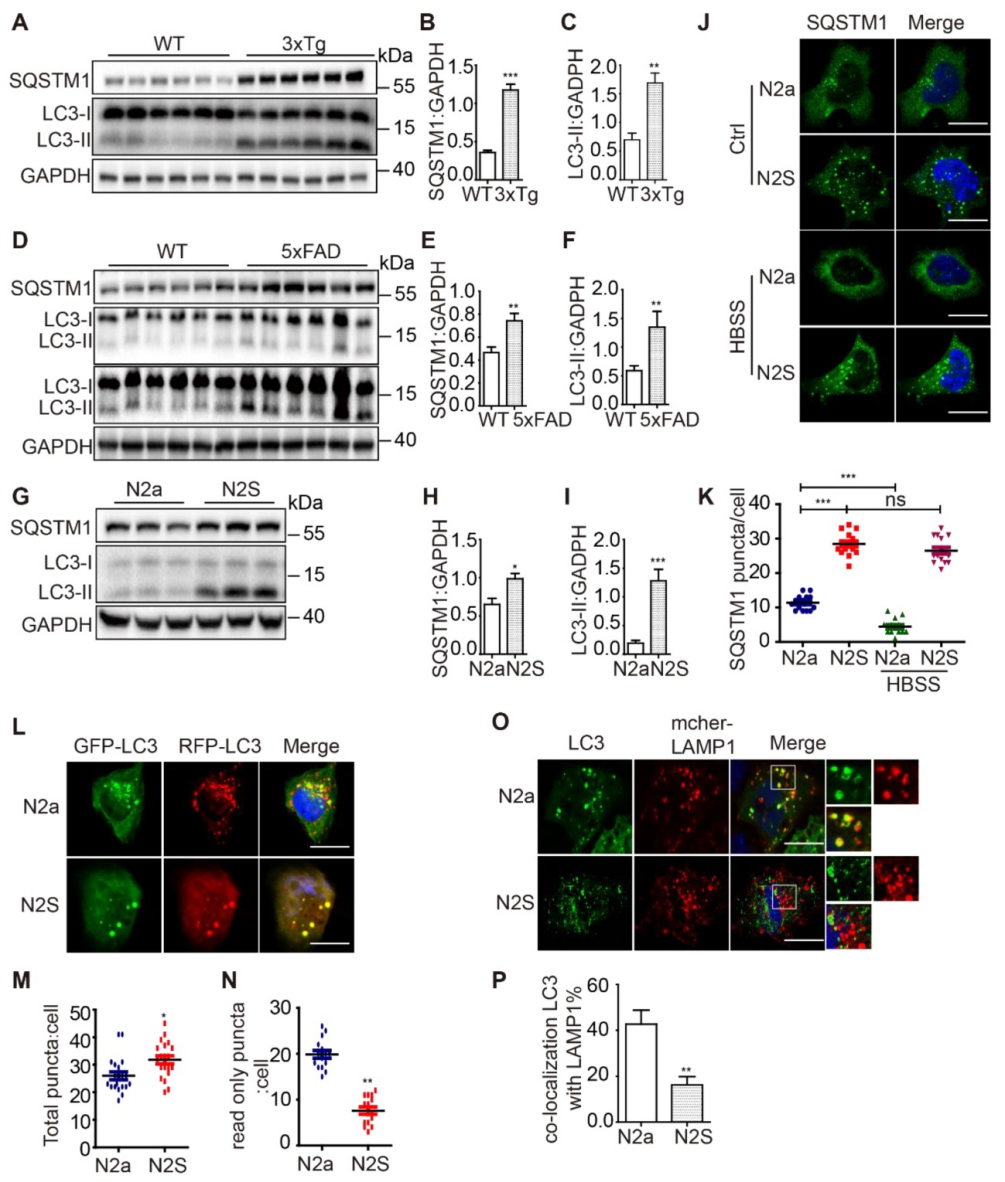
AD cell and mice models exhibit inhibition of autophagosome maturation. (**A**) Immunoblotting was used to detect the expression of LC3 and SQSTM1 in the hippocampus region of 12-month-old C57BL/6, 3xTg AD mice. (**C-B**) Quantitative data in panel Figure 1A. Data are quantified as mean ± SEM (n = 3). *P < 0.05, **P < 0.01, ***P < 0.001 *vs.* the relative control. (**D**) Immunoblotting was used to detect the expression of LC3 and SQSTM1 in the hippocampus region of 12-mo-old 5xFAD AD mice. (**E-F**) Quantitative data in panel Figure 1D. Data are quantified as mean ± SEM (n =3). *P < 0.05, **P < 0.01, ***P < 0.001 *vs.* the relative control. (**G**) Immunoblotting was used to detect the expression of LC3 and SQSTM1/p62 in the APP over-expressed N2S cells. (**H-I**) Quantitative data in panel Figure 1G. Data are quantified as mean ± SEM (n = 3). *P < 0.05, **P < 0.01, ***P < 0.001 *vs.* the relative control. (**J-K**) SQSTM1 levels were determined by immunofluorescence. Quantification data are presented as the mean ± SEM, n = 20-25 cells from 3 independent experiments. ***P < 0.001 *vs.* the relative control, ns, not significant. Scale bar, 7.5 μm. (**L-N**) Autophagosome maturation in N2a and N2S cells was determined by the GFP-RFP-LC3 probe. Quantification data are presented as the mean ± SEM, n = 20-25 cells from 3 independent experiments. Scale bar, 7.5 μm. (**O-P**) N2a and N2S cells were transiently transfected with GFP-*Lc3* and mCherry-LAMP1, the colocalization of autophagosomes and lysosomes was visualized under confocal microscope. Quantification data are presented as the mean ± SEM, n = 20-25. *P < 0.05, **P < 0.01, ***P < 0.001 *vs.* the relative control. Scale bar, 7.5 μm.

Autophagy dysfunction has been observed in the brain tissue of AD patients as well as in several AD models 20, 21. To provide a comprehensive analysis of the autophagic activity in both Aβ- and P-tau-based AD models, we examined the autophagosome maturation process in N2a mouse neuroblastoma cells stably expressing the human Swedish mutant APP695 (N2S cells) (**Figure S1A**), in a 3xTg AD mice (APP Swedish, MAPT P301L, and PSEN1 M146V) model (**Figure S1B**) and in a 5xFAD model (M146L and L286V mutations in PSEN1, and Swedish (K670N/M671L), Florida (I716V), and London (V717I) mutations in APP) (**Figure S1C**). We found that both LC3-II and SQSTM1 accumulation in 3xTg AD mice (**Figure 1A-C**), 5xFAD mice (**Figure 1D-F**) and N2S cells (**Figure 1G-I**). Immunofluorescence staining showed massive accumulation of SQSTM1 in N2S cells. Induction of autophagy by HBSS reduced SQSTM1 puncta effectively in N2a cells but less efficiently in N2S cells (**Figure 1J-K**). Interestingly, almost all SQSTM1 puncta co-localized with LC3 puncta in N2S cells (**Figure S1D-E**), indicating the inhibition of autophagic degradation. The RFP-GFP-LC3 probe has been widely used to monitor autophagy flux 22, based on the fact that GFP is more rapidly quenched than RFP in acidic environments in lysosomes. We used the RFP-GFP-LC3 probe to monitor autophagy flux in N2S cells and found that the number of yellow puncta (both GFP- and RFP-positive) increased (**Figure 1L-M**) while the number of red-only puncta decreased in N2S cells (**Figure 1N**). Meanwhile, we found that colocalization of autophagosome marker LC3 and lysosome marker LAMP1was decreased in N2S cells (**Figure 1O-P**). Taken together, these findings indicate that autophagosome maturation is impaired in AD cell and mouse models.

### Active form of RAB7 in autophagosome fractions and MON1A-CCZ1 GEF activity are decreased in AD cell and mice models

**Figure 2:**
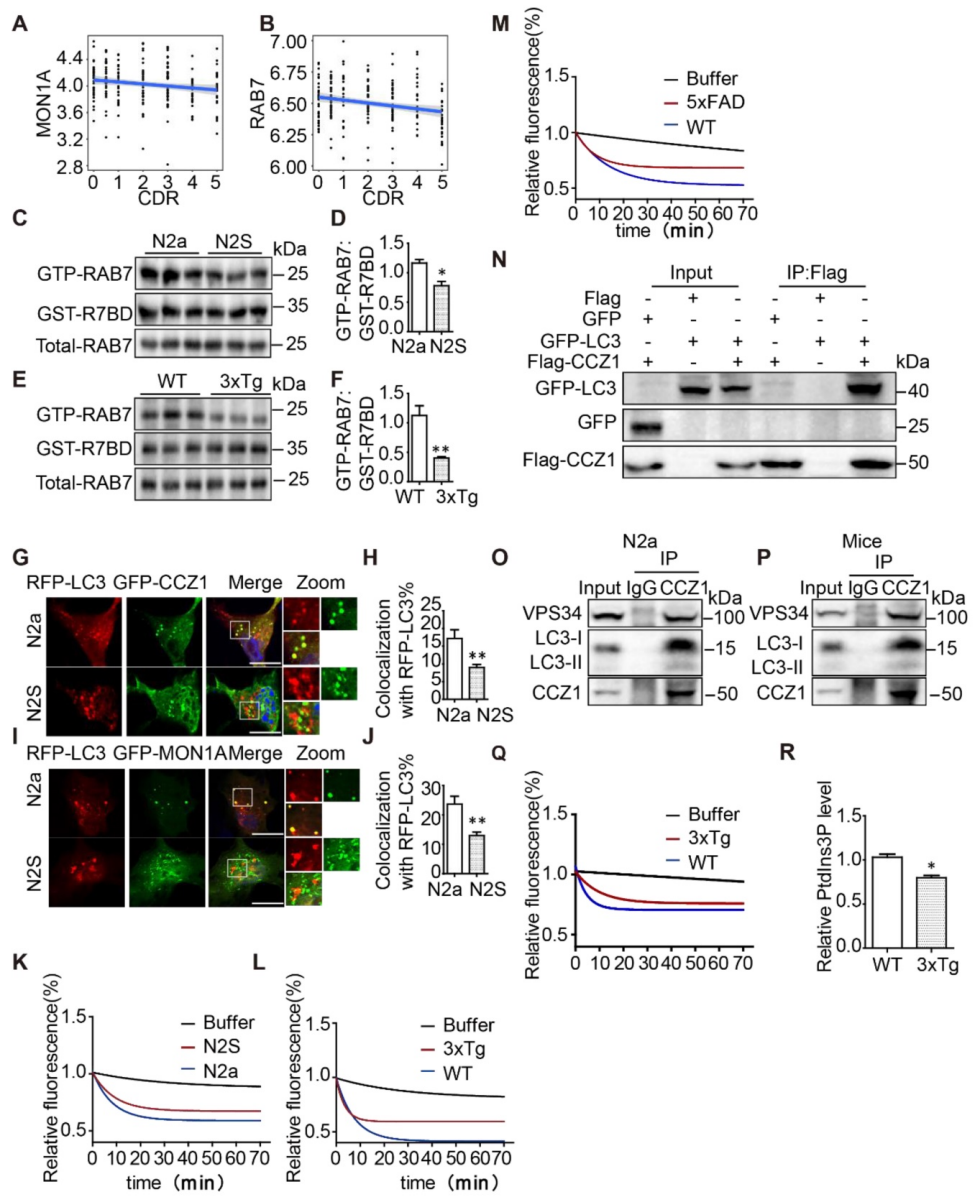
Active form of RAB7 in autophagosome fractions and MON1A-CCZ1 GEF activity are decreased in AD cell and mice models. (**A-B**) Bioinformatics analysis of correlation between expression levels of MON1A and RAB7 mRNA and CDR score in parahippocampal gyrus regions of AD patients and controls from the Mount Sinai Brain Bank (MSBB) cohort. (**C-D**) GTP-RAB7 in autophagosomes isolated from N2a and N2S cells were determined by GST-R7BD affinity-isolation assay. Data are quantified as mean ± SEM (n = 3). *P < 0.05, **P < 0.01, *vs.* the relative control. (**E-F**) GTP-RAB7 in autophagosomes isolated from WT and 3xTg AD mice were determined by GST-R7BD affinity-isolation assay. Data are quantified as mean ± SEM (n =3). *P < 0.05, **P < 0.01, *vs.* the relative control. (**G-H**) N2a and N2S cells were transiently transfected with RFP-*Lc3* and GFP-CCZ1. Colocalization of RFP-LC3 and GFP-CCZ1 was visualized under confocal microscope. Quantification data are presented as mean ± SEM, n = 20. *P < 0.05, **P < 0.01, *vs.* the relative control. Scale bar, 7.5 μm. (**I-J**) N2a and N2S cells were transiently transfected with RFP-*Lc3* and GFP-MON1A. The colocalization of RFP-LC3 and GFP-MON1A was visualized under confocal microscope. Quantification data are presented as the mean ± SEM, n = 20. *P < 0.05, **P < 0.01, ***P < 0.001, *vs.* the relative control. Scale bar, 7.5 μm. (**K**) CCZ1-MON1A protein was purified by CCZ1 antibody from N2a or N2S cells, and was then subjected to the GEF assay. (**L-M**) CCZ1-MON1A protein was purified by CCZ1 antibody from hippocampus of 3xTg AD mice or 5xFAD AD mice, and was then subjected to the GEF assay. (**N**) N2a cells were transiently transfected with Flag/Flag-CCZ1 and GFP-*Lc3*, followed by immunoprecipitation (IP) with anti-Flag antibody; IP products were resolved by SDS-PAGE and analyzed by immunoblotting with the corresponding antibodies. (**O-P**) N2a cells and mice brain tissue lysates were subjected to immunoprecipitation using CCZ1 antibody, and the IP products were resolved by SDS-PAGE and analyzed by immunoblotting with the LC3 and VPS34 antibodies (**Q**) VPS34-associated CCZ1-MON1A protein was purified by VPS34 antibody from WT or 3xTg AD mouse, then was subjected to the GEF assay. (**R**) CCZ1-binding VPS34 was purified by CCZ1 antibody from WT and 3xTg AD mice brain, and was subjected to lipid kinase activity assay.

Our and others previous studies have revealed the critical role of RAB7 in regulating autophagosome maturation 19. To investigate how genes involved in the CCZ1-MON1A-RAB7 complex are affected in AD, we examined their transcriptional changes in four brain regions of AD patients and controls from the Mount Sinai Brain Bank (MSBB) cohort (PMID) 23. Both MON1A (Rho = -0.19, adj.p = 0.01) and RAB7 (Rho = -0.22, adj.p = 0.003) mRNA expression demonstrated a negative correlation with CDR score only in the parahippocampal gyrus (BM36) which is particularly vulnerable to AD (**Figure 2A**-**B**), but not in other brain regions, indicating a region-specific impact of AD on these genes. CCZ1 expression was not significantly affected by AD in any of the four brain regions. The data suggest progressive declines of CCZ1-MON1A GEF activity and of the active form of RAB7 might occur during the course of AD in the parahippocampal gyrus region.

To understand whether RAB7 activity is impaired in AD cell and mouse models, we applied the GST-tagged RAB7-binding domain (GST-R7BD) of RILP 19, 24 and GTP beads 19, 25, which selectively bind GTP-binding proteins, to monitor the level of the GTP form of RAB7 (GTP-RAB7) in autophagosome fractions (**Figure S1F**). We found that GST-R7BD pulled down much less GTP-RAB7 in autophagosome fractions isolated from N2S cells and from the hippocampus tissue of 3xTg AD mice, compared with that isolated from N2a cells and WT mice (**Figure 2C-F**), without affecting the total RAB7 protein level. Consistently, GTP beads also pulled down less GTP-RAB7 in autophagosome fractions isolated from N2S cells and hippocampus tissue of 3xTg AD mice (**Figure S1G-J**). Interestingly, the total amount of GTP-RAB7 increased rather than decreased in N2S cells and hippocampus tissue of 3xTg mice (**Figure S1K-N**), indicating impaired autophagosome maturation may trigger a compensation mechanism in AD models.

CCZ1-MON1A complex is the GEF for RAB7 and has been reported to interact with Atg8 (mammalian homolog LC3) and to localize on autophagosomes in yeast 18. We confirmed that the CCZ1-MON1 complex co-localizes with autophagosome marker LC3 in N2a cells (**Figure 2G-J**) and interacts with LC3 in N2a cells and mice brain tissue (**Figure 2N-P**). To understand whether reduced GTP-RAB7 on autophagosomes is due to impaired CCZ1-MON1 complex function, firstly, we determined the localization of CCZ1-MON1 complex on autophagosomes in N2S cells by confocal microscopy. Secondly we immune-purified the CCZ1-MON1 complex from N2S cells, 3xTg mice and 5XFAD mice models to perform in vitro GEF assays 17, 19. As expected, colocalization between GFP-CCZ1/GFP-MON1A and RFP-LC3 was reduced in N2S cells (**Figure 2G-J**), and the CCZ1-MON1 complex GEF activity was decreased in N2S cells (**Figure 2K**), 3xTg mice (**Figure 2L**) and 5XFAD mice (**Figure 2M**) models.

Our previous work revealed that CCZ1 interacts with PIK3C3, which produces PtdIns3P to regulate autophagosome formation and maturation 19. PtdIns3P has been reported to recruit the CCZ1-MON1A complex to regulate RAB7 activation 26. We wondered whether VPS34-associated CCZ1-MON1A GEF activity was reduced in AD mouse models. CCZ1-MON1A protein immunopurified by VPS34 antibody was used to perform the GEF assay. From the results we can see PIK3C3-associated CCZ1-MON1A GEF activity was markedly decreased in 3xTg AD mouse brain tissue (**Figure 2Q**). Consistently, the CCZ1-associated PIK3C3 kinase activity is reduced in 3xTg AD mice (**Figure 2R**). Collectively, the above results indicate that the active form of RAB7 (GTP-RAB7) is reduced specifically on the autophagosomes in AD cell and mice models due to impaired CCZ1-MON1A GEF activity.

### CCZ1-MON1A is activated during autophagy and positively regulates autophagosome maturation

**Figure 3:**
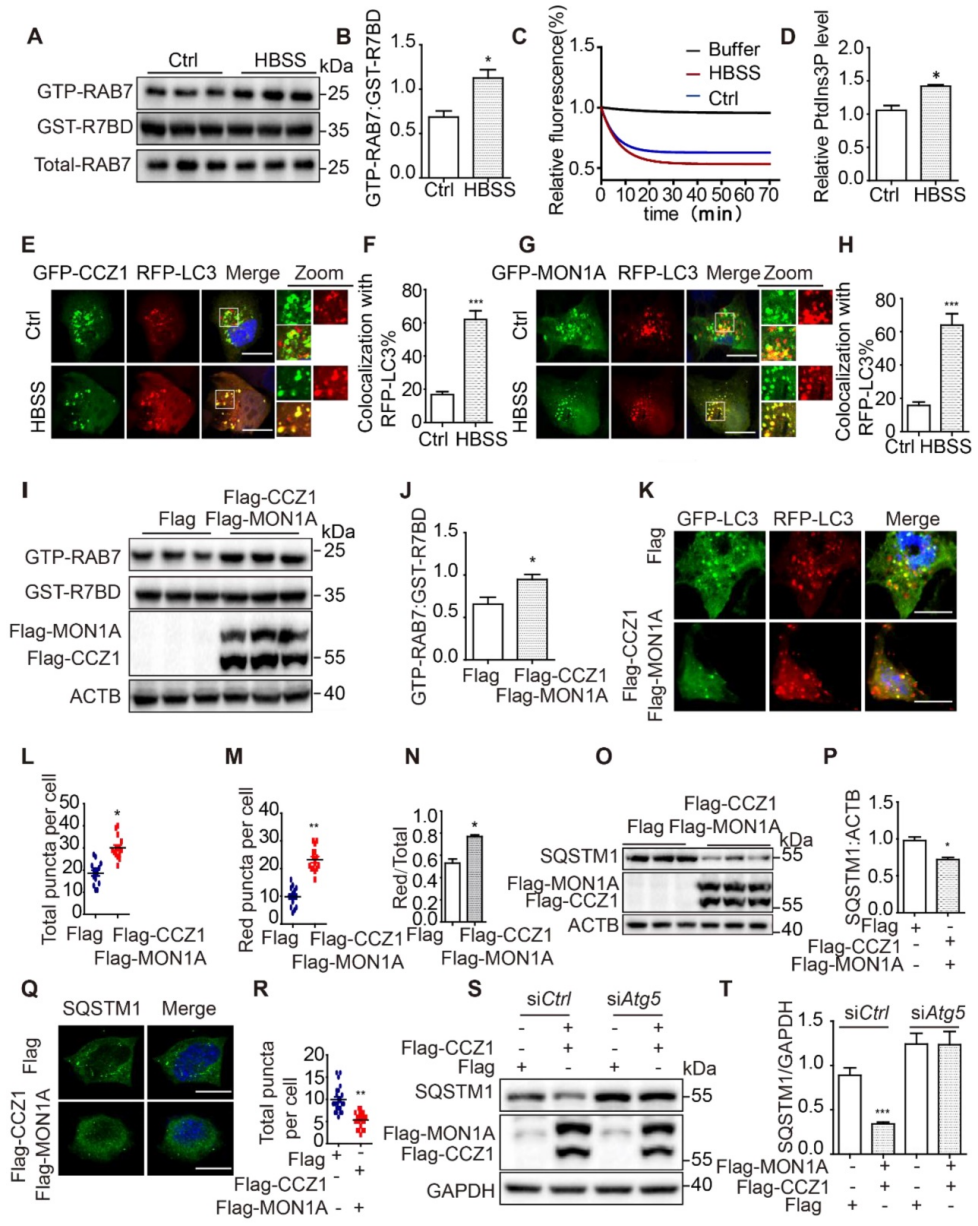
CCZ1-MON1A was activated during autophagy and positively regulates autophagosome maturation. (**A-B**) GTP-RAB7 in autophagosomes isolated from normal and HBSS-treated N2a cells was determined by GST-R7BD affinity-isolation assay. (**C**) PIK3C3-associated GEF activity was increased in a starvation-induced autophagy condition. (**D**) Quantification of CCZ1-IPed PIK3C3 kinase activity in normal and HBSS-treated cells. (**E-F**) N2a cells were transiently transfected with GFP-CCZ1 and RFP-*Lc3*. Colocalization of CCZ1 and the LC3 under normal or HBSS-treated conditions were visualized under confocal microscope. Quantification data are presented as the mean ± SEM, n = 20-25 cells from 3 independent experiments. *P < 0.05, **P < 0.01, *vs.* the relative control. Scale bar: 5 μm. (**G-H**) N2a cells were transiently transfected with GFP-MON1A and RFP-*Lc3*. Colocalization of GFP-MON1A and the RFP-LC3 under normal or HBSS-treated conditions were visualized under confocal microscope. Quantification data are presented as the mean ± SEM, n = 20-25 cells from 3 independent experiments. *P < 0.05, **P < 0.01, *vs.* the relative control. Scale bar: 5 μm. (**I-J**) GTP-RAB7 in N2a cell was determined by GST-R7BD affinity-isolation assay. Data are quantified as mean ± SEM (n = 3). *P < 0.05, **P < 0.01, *vs.* the relative control. (**K-N**) Autophagosome maturation in N2a and N2a cells over-expressing CCZ1-MON1A was determined by GFP-RFG-LC3 probe. Quantification data are presented as the mean ± SEM, n = 20-25 cells from 3 independent experiments. *P < 0.05, **P < 0.01, *vs.* the relative control. Scale bar: 5 μm. (**O** and **P**) SQSTM1 levels were determined by immunoblotting after over-expression of CCZ1-MON1A. *P < 0.05, **P < 0.01, ***P < 0.001 *vs.* the relative control. (**Q** and **R**) SQSTM1 levels were determined by immunofluorescence after over-expression of CCZ1-MON1A. Quantification data are presented as the mean ± SEM, n = 20-25 cells from 3 independent experiments. *P < 0.05, **P < 0.01, *vs.* the relative control. Scale bar: 5 μm. (**S** and **T**) The N2a cells over-expressing CCZ1-MON1A were transfected with control SiRNA or Atg5 SiRNA. The levels of SQSTM1 were examined by immunoblotting. Data are quantified as mean ± SEM (n =3). *P < 0.05, **P < 0.01, *vs.* the relative control.

Our data show that CCZ1-MON1A puncta partially co-localized with autophagic structures. However, the effect of the CCZ1-MON1A complex on autophagy has not been fully explored. By using GST-R7BD affinity-isolation assay as described previously, we found that GTP-RAB7 was significantly increased on autophagosomes in starvation-induced autophagy conditions, without affecting total RAB7 protein level (**Figure 3A-B**). PIK3C3-associated GEF activity and CCZ1-associated VPS34 kinase activity both increased in starvation-induced autophagy conditions (**Figure 3C-D**). More interestingly, we found that co-localization between CCZ1-MON1A and LC3 was increased in starvation-induced autophagy conditions (**Figure 3E-H**). To understand the effect of CCZ1-MON1A on autophagy, we over-expressed CCZ1-MON1A in N2a cells and observed increased GTP-RAB7 as expected (**Figure 3I-J**). We applied an RFP-GFP-LC3 probe to examine autophagic flux in CCZ1-MON1A over-expression cells (**Figure 3K**). Compared with the empty vector group, CCZ1-MON1A over-expression led to increase of yellow puncta (**Figure 3L**), of red-only puncta (**Figure 3M**), and of the ratio of red-only puncta to yellow puncta (**Figure 3N**), indicating the enhancement of autophagosome maturation.

To confirm the function of CCZ1-MON1A in regulating autophagic degradation in cells, we examined the levels of the autophagy substrate SQSTM1 in N2a (**Figure 3O**) and N2S cells (**Figure S2A**). We found that CCZ1-MON1A over-expression significantly reduced SQSTM1 protein levels and SQSTM1 puncta in N2a and N2S cell (**Figure 3O-R, Figure S2A-D**). At the same time, we showed that over-expression of CCZ1 or MON1A alone did not affect SQSTM1 level (**Figure S2E**). Finally, we confirmed that the CCZ1-MON1A over-expression-mediated degradation of SQSTM1 is dependent on autophagy as the effect can be blocked by Atg5 SiRNA transfection or CQ treatment (**Figure 3S-T, Figure S2F-G**).

**Figure 4:**
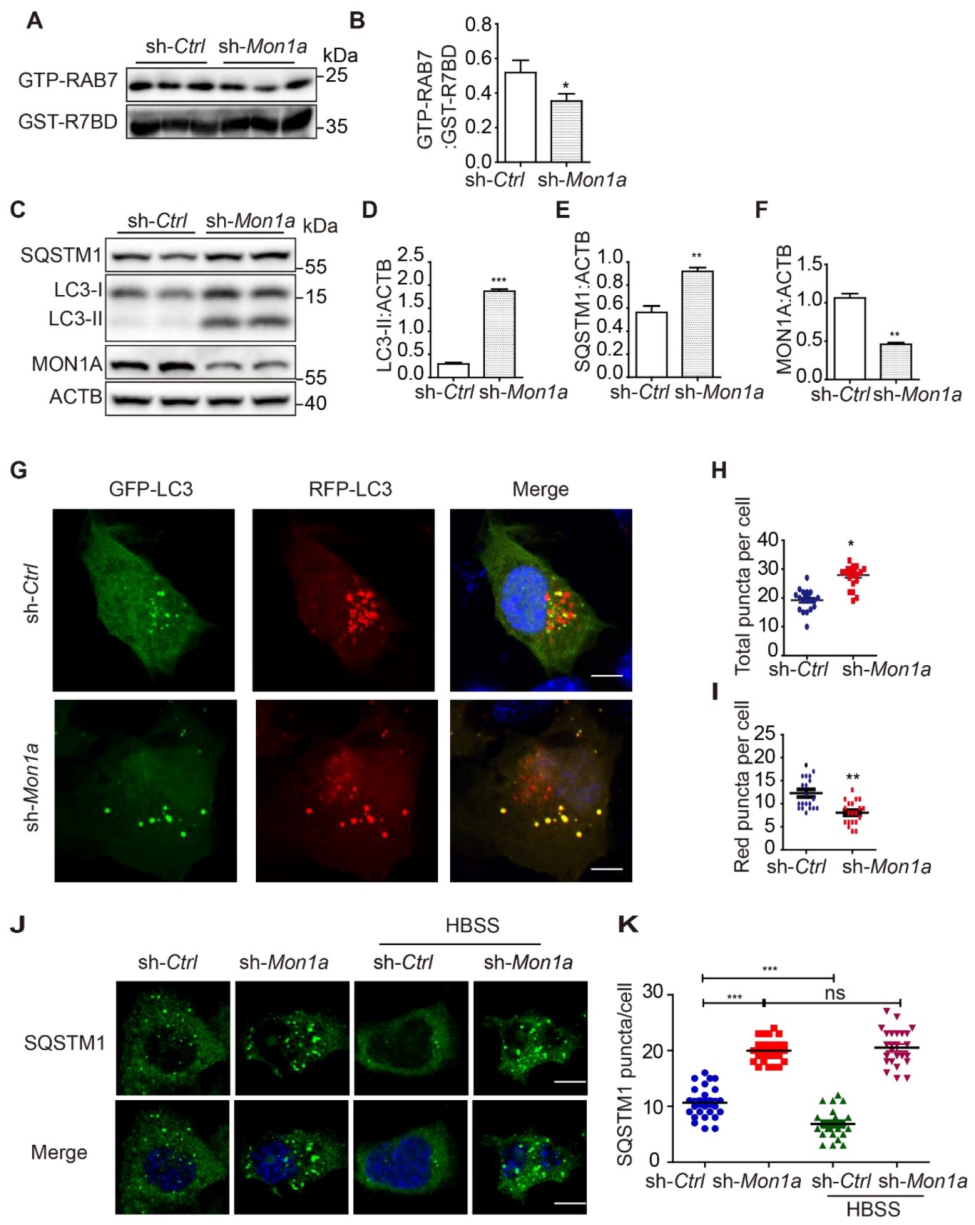
(**A-B**) N2S cells were transfected with *Mon1a* shRNA or nontargeting shRNA. The level GTP-RAB7 were detected by GST-R7BD affinity-isolation assay. Data are quantified as mean ± SEM (n = 3). *P < 0.05, **P < 0.01, ***P < 0.001 *vs.* the relative control. (**C-F**) Cells were transfected with *Mon1a* shRNA, and the levels of SQSTM1 and LC3-II were examined by immunoblotting. *P < 0.05, **P < 0.01, ***P < 0.001 *vs.* the relative control. (**G-I**) Autophagosome maturation in N2a cells transfected with *Mon1a* shRNA or nontargeting shRNA were determined by the GFP-RFG-LC3 probe. Quantification data are presented as the mean ± SEM, n = 20-25 cells from 3 independent experiments. *P < 0.05, **P < 0.01, *vs.* the relative control. Scale bar: 5 μm. (**J-K**) SQSTM1 levels were determined by immunofluorescence in control and *Mon1a* KD cells. Quantification data are presented as the mean ± SEM, n = 20-25 cells from 3 independent experiments. *P < 0.05, **P < 0.01, *vs.* the relative control. Scale bar: 5 μm.

In addition, we knocked down (KD) MON1A expression in N2a cells by using *Mon1a* shRNA and found that *Mon1a* KD significantly decreased GTP-RAB7 level and increased LC3-II and SQSTM1 levels (**Figure 4A-F**). Autophagosome maturation in N2a and Mon1a KD N2a cells was determined by the RFP-GFP-LC3 (**Figure 4G**). The number of total puncta increased (**Figure 4H**) while the number of red-only puncta (**Figure 4I**) decreased in Mon1a KD cells. Immunofluorescence staining showed the number of SQSTM1 puncta significantly increased in Mon1a KD N2a cells (**Figure 4J-K**). We enhanced autophagy flux by starving cells with HBSS which enhanced the degradation of SQSTM1 in N2a cells but not in N2a KD Mon1a cells (**Figure 4J-K**). Taken together, these results demonstrate that CCZ1-MON1A positively regulates autophagosome maturation in neuronal cells.

### CCZ1-MON1A positively regulates autophagic degradation of APP-CTFs, Aβ and P-tau

**Figure 5:**
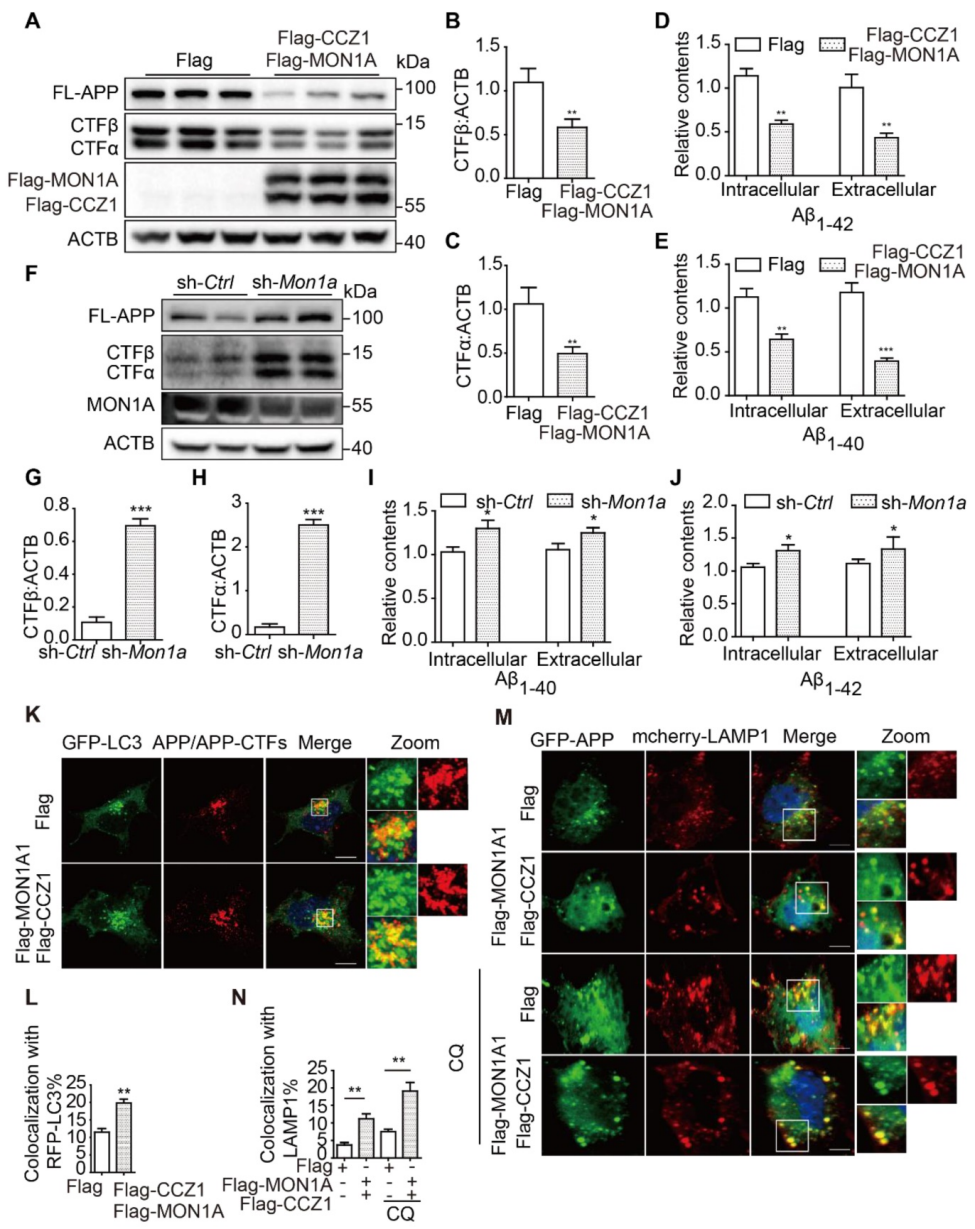
(**A-C**) After the N2S cells were transfected with Flag-CCZ1/Flag-MON1A or Flag vehicle, the levels of FL-APP, APP-CTFs and Flag-CCZ1, Flag-MON1A were examined by immunoblotting. Data are quantified as mean ± SEM (n = 3). *P < 0.05, **P < 0.01, *vs.* the relative control. (**D-E**) Intracellular and extracellular Aβ1-40 and Aβ1-42 levels in N2S cells over-expressing CCZ1-MON1A were determined by ELISA analysis. Data are quantified as mean ± SEM (n = 3). *P < 0.05, **P < 0.01, ***P < 0.001 *vs.* the relative control. (**F-H**) N2S cells were transfected with *Mon1a* shRNA or nontargeting shRNA. The levels of FL-APP, APP-CTFs were detected by immunoblotting. Data are quantified as mean ± SEM (n = 3). *P < 0.05, **P < 0.01, ***P < 0.001 *vs.* the relative control. (**I-J**) ELISA analysis of intracellular and extracellular Aβ levels in control and *Mon1a* KD cells. Data are quantified as mean ± SEM (n = 3). *P < 0.05, **P < 0.01, ***P < 0.001 *vs.* the relative control. (**K-L**) N2S cells over-expressing CCZ1-MON1A were transiently transfected with GFP-*Lc3* and stained with APP antibody. The colocalization of GFP-LC3 and APP/APP-CTFs was visualized under confocal microscope. Quantification data are presented as the mean ± SEM, n = 20-25 cells from 3 independent experiments. Scale bar, 7.5 μm. (**M-N**) N2S cells over-expressing CCZ1-MON1A were transiently transfected with mcherry-LAMP1 and the GFP-APP, and the colocalization of mcherry-LAMP1 and GFP-APP under basal or CQ treated conditions were visualized under confocal microscope. Quantification data were presented as the mean ± SEM, n = 20-25 cells from 3 independent experiments. Scale bar, 7.5 μm.

To understand whether impaired CCZ1-MON1A-RAB7 module function is responsible for the accumulation of AD-related protein APP-CTFs, Aβ and P-tau in AD models, we overexpressed CCZ1-MON1A complex in N2S cells. We found that over-expression of CCZ1-MON1A significantly reduced APP-CTF (**Figure 5A-C**) and Aβ levels (**Figure 5D-E**). Meanwhile, we examined the effect of CCZ1-MON1A over-expression on inducible HEK 293 cells expressing pTRE3G-mcherry-BI promoter-EGFP Tau P301L (HEK 293 3G-EGFP-Tau P301L/mcherry) and found that over-expression of CCZ1-MON1A significantly reduced the GFP-P-tau level (**Figure S3A-D**). To further confirm the function of CCZ1-MON1A in APP-CTFs degradation and Aβ production, we generated *Mon1a* knockdown (KD) N2S cell using shRNA*-Mon1a* (**Figure 5F**). KD of *Mon1a* led to dramatic accumulation of APP-CTFs and Aβ in N2S cells (**Figure 5F-J**). These results indicate that CCZ1-MON1A is an important determinant of APP-CTFs, Aβ and P-tau contents. Interestingly, the increase of CTFs levels are more dramatic than Aβ levels in *Mon1a* knockdown (KD) N2S cells, suggesting that there is a difference in the fate of the two catabolites and that additional proteases may target accumulated Aβ for degradation.

To better understand how CCZ1-MON1A mediates autophagy and promotes APP-CTFs degradation, we examined whether CCZ1-MON1A would recruit APP/APP-CTFs into autophagosomes and lysosomes, as previous data showed that CCZ1-MON1A interacts with both LC3 (**Figure 2N-P**) and APP 19. As the results show, the co-localization of APP/APP-CTFs with autophagosome marker LC3 and lysosome marker LAMP1 was significantly increased in CCZ1-MON1A-overexpressed cells (**Figure 5K-N**). Interestingly, over-expression of CCZ1-MON1A significantly decreased the percentage of APP/APP-CTFs present in the RAB5-positive early endosomes while it increased distribution of APP/APP-CTFs in the RAB7-positive late endosomes (**Figure S4A-D**), indicating that CCZ1-MON1A also promoted endocytic maturation of APP/APP-CTFs. These results suggest that over-expression CCZ1-MON1A promotes recruitment of APP and APP-CTFs into autophagic-lysosome structures.

### CCZ1-MON1A regulates APP-CTFs and Aβ levels through autophagy

**Figure 6:**
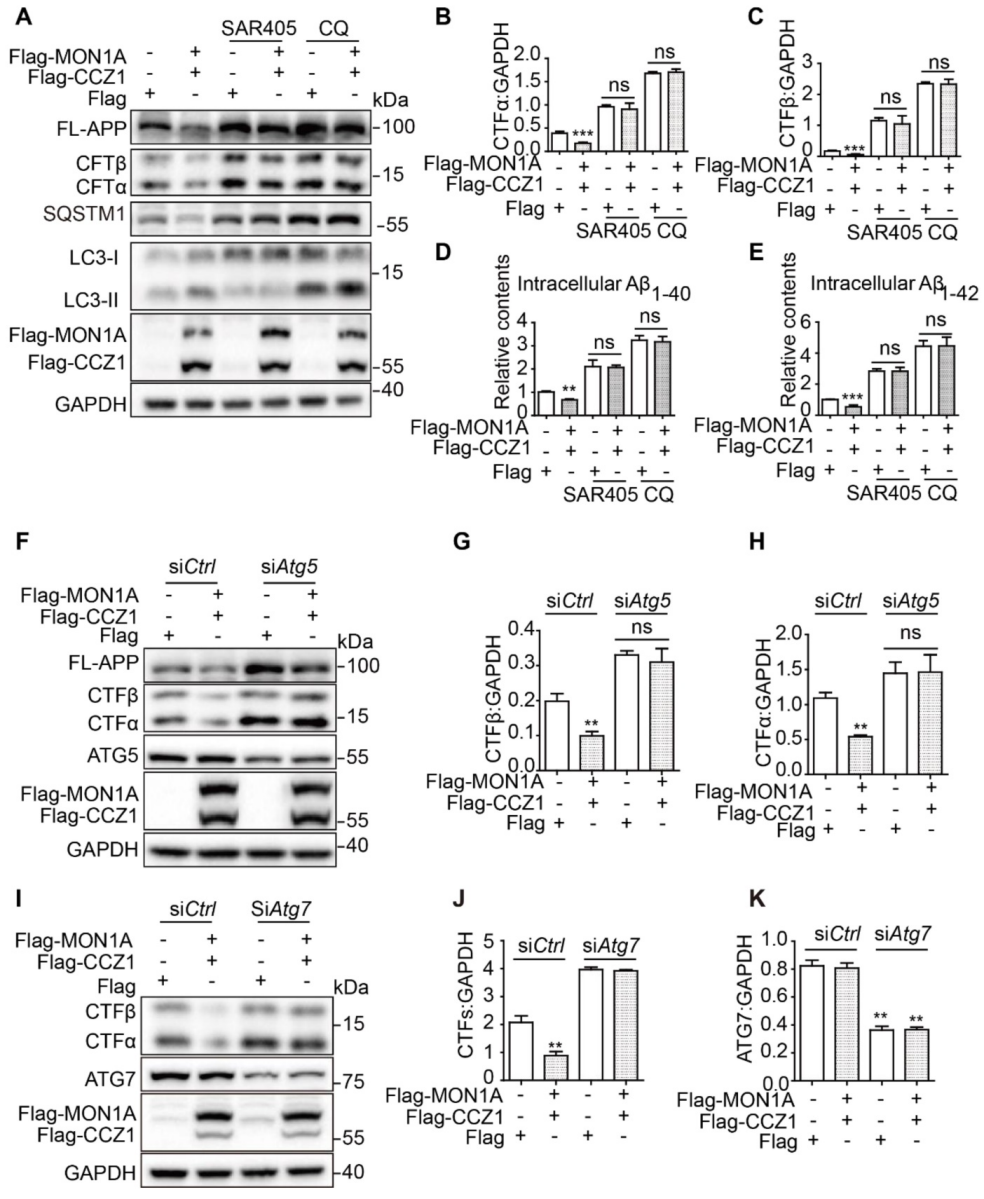
CCZ1-MON1A regulates APP-CTFs and Aβ levels through autophagy. (**A**) N2S cells were transfected with Flag-CCZ1/Flag-MON1A in the presence or absence of SAR405 and CQ. The levels of FL-APP and APP-CTFs were examined by immunoblotting. (**B-C**) Quantitative results of Figure 6A show that SAR405 and CQ blocked the CCZ1-MON1A over-expression-mediated reduction of APP-CTFs. *P < 0.05, **P < 0.01, ***P < 0.001 *vs.* the relative control. (**D-E**) CCZ1-MON1A over-expressing N2S cells were treated with SAR405 or CQ, and the intracellular Aβ1-40 and Aβ1-42 levels were examined by ELISA. *P < 0.05, **P < 0.01, ***P < 0.001 *vs.* the relative control. (**F-H**) N2S cells were transfected with control or *Atg5* siRNA 48h before being transfected with CCZ1-MON1A plasmids. FL-APP and APP-CTFs levels were determined by immunoblotting. Data are quantified as mean ± SEM (n = 3). *P < 0.05, **P < 0.01, *vs.* the relative control. (**I-K**) N2S cells were transfected with control or *Atg7* siRNA 48h before being transfected with CCZ1-MON1A plasmids. APP-CTFs levels were determined by immunoblotting. Data are quantified as mean ± SEM (n = 3). *P < 0.05, **P < 0.01 *vs.* the relative control.

We and others have found that enhanced autophagy promotes Aβ degradation 27. Our previous data showed that CCZ1-MON1A is a positive regulator of autophagy and promotes degradation of APP-CTF and Aβ. To determine whether CCZ1-MON1A-mediated APP-CTF degradation is dependent on autophagy, we treated CCZ1-MON1A over-expression cells with chloroquine (CQ) and SAR405 to inhibit autophagy (**Figure 6A-E**). We found that the CCZ1-MON1A over-expression-mediated reductions of APP-CTFs and Aβ were abolished by CQ and SAR405 treatment in N2S cells (**Figure 6A-E**). Furthermore, KD of *Atg5* and *Atg7*, two important proteins for autophagosome initiation, blocked CCZ1-MON1A over-expression-induced APP-CTFs degradation (**Figure 6F-K**). These results indicate that autophagy is required for CCZ1-MON1A-mediated degradation of APP-CTFs and Aβ.

### AAV-mediated CCZ1-MON1A over-expression in the hippocampus alleviated autophagy impairment and AD-related behavioral and neuropathological changes in 3xTg AD mice

To confirm the effects of CCZ1-MON1A in regulating AD-related behavioral and neuropathological changes, AAV9-Flag-ccz1 /AAV9-Flag-mon1a (AAV-CM) or empty vector AAV-FLAG (AAV-VE) were stereotaxically injected into both sides of the hippocampus of 3xTg AD mice and WT C57 mice 28. There were nine mice in each group. Two months after injection, the mice were subjected to spatial memory testing, and their brains were used for biochemical and neuropathological analyses. Immunostaining of the brain slices revealed that injection of the virus into the DG regions led to highly restricted hippocampal expression (**Figure S5A-D**).

**Figure 7:**
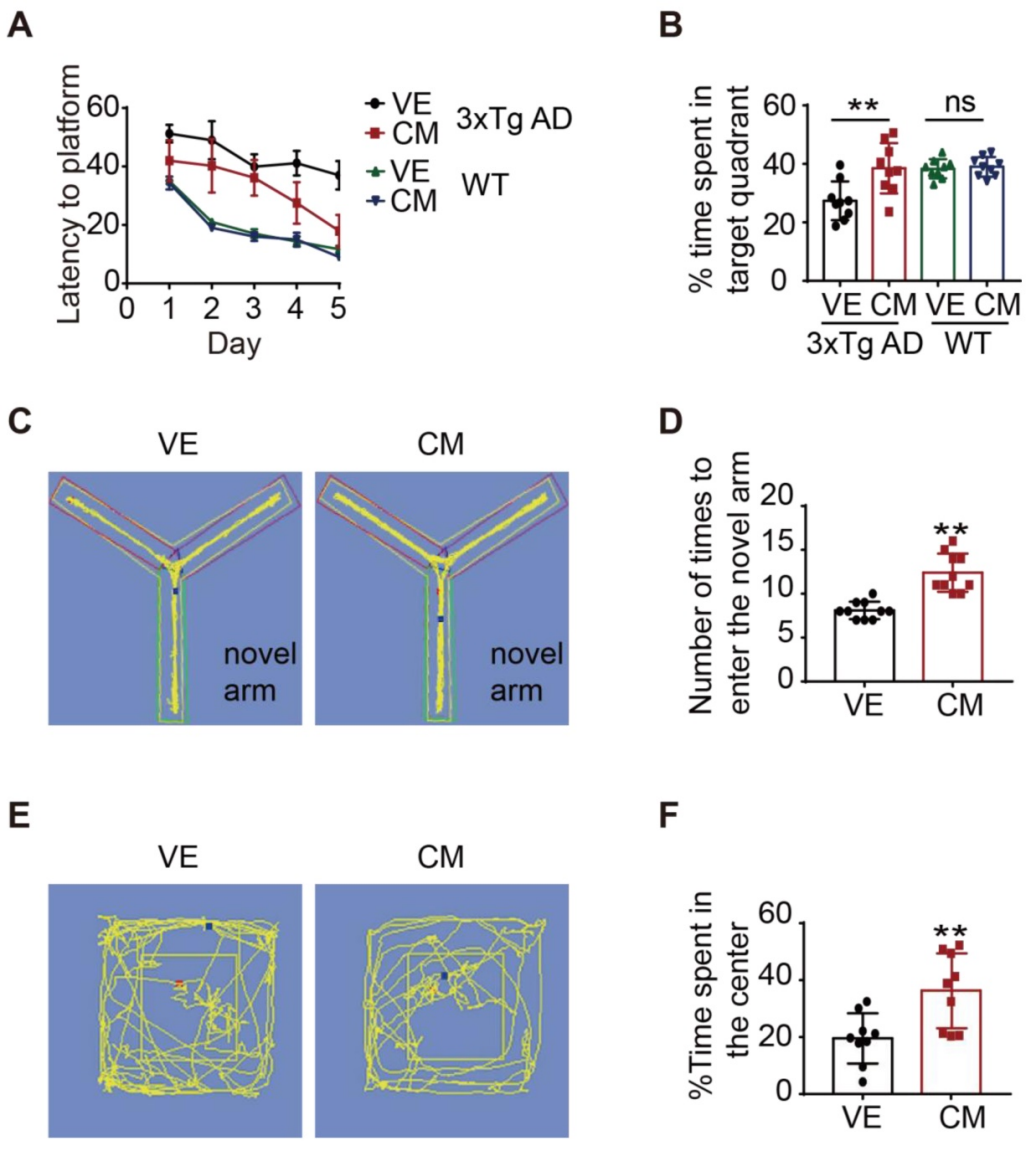
CCZ1-MON1A improves exploratory behavior, spatial learning, and memory acquisition in 3xTg mice. (**A-B**) The spatial memory of WT or 3xTg mice receiving AAV injection was evaluated by morris water maze (MWM). Training trials (60 sec each) were performed 4 times a day for 5 days and the time for mice to find the platform was recorded. After 5 days training, the platform was removed and the time that mice stay in the platform quadrant was recorded. Data are quantified as mean ± SEM (n = 9). *P < 0.05, **P < 0.01 *vs.* the relative control, ns, not significant. (**C-D**) Short-term memory was test by Y-maze. The time that the mice spent in new arm was recorded. Data are quantified as mean ± SEM (n = 9). *P < 0.05, **P < 0.01 *vs.* the relative control. (**E-F**) The anxiety behavior was measured by open field test. The time that the mice spent in the center of the box was recorded. Data are quantified as mean ± SEM (n = 9). *P < 0.05, **P < 0.01 *vs.* the relative control.

The Morris water maze test (MWM) 29 was used to evaluate the spatial memory of 3xTg mice receiving AAV-CM or AAV-FLAG injections. The 3xTg AD mice receiving AAV-CM injections travelled a significantly shorter time to find the hidden platform compared with those receiving AAV-Flag treatment, while WT mice receiving AAV-CM injections did not display any alternation in the time to find the hidden platform (**Figure 7A**). In the probe trial, the CM-AAV-injected 3xTg AD mice spent a significantly greater percentage of time in the target quadrant when compared to the vehicle-treated 3xTg mice (**Figure 7B**). While the CM-AAV-injected WT mice showed the similar time spent in the target quadrant compared to the vehicle-treated WT mice (**Figure 7B**). We next investigated short-term memory using the Y-maze text 30. 3xTg AD mice receiving CM-AAV injection more frequently entered the novel arm and spent more time there (**Figure 7C-D**). These results suggest that over-expression of CCZ1-MON1A in the hippocampus selectively improved cognitive function of 3xTg AD mice.

Anxiety is one of the clinical features of AD patients, and 3xTg AD mice also display anxious behavior 31. We used an open field test to test whether CCZ1-MON1A over-expression might exert anxiolytic effects. We found that over-expression of CCZ1-MON1A significantly increased the time in the center area (**Figure 7E-F**). This data suggested that over-expression of CCZ1-MON1A decreased anxiety in 3xTg AD mice.

**Figure 8:**
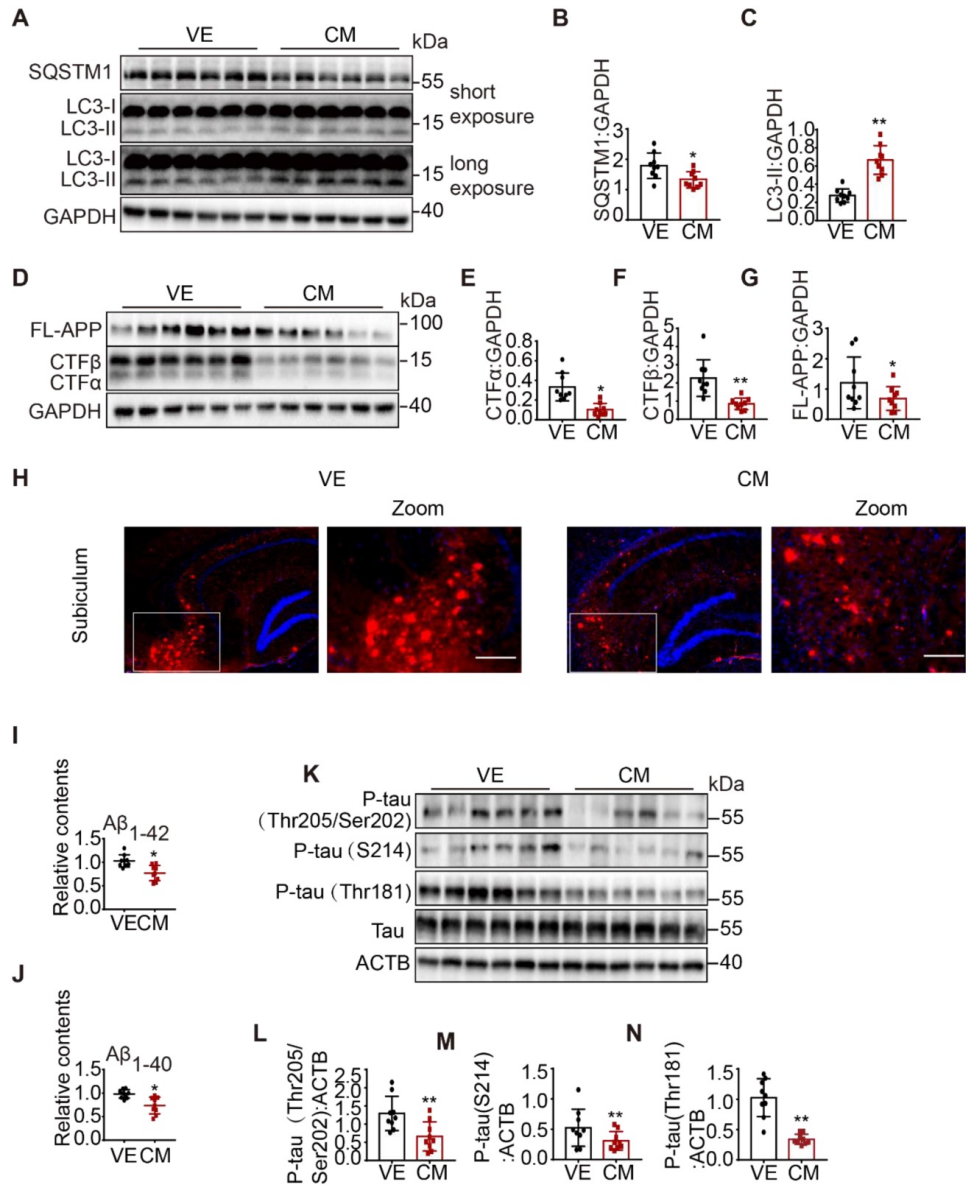
AAV-mediated CCZ1-MON1A over-expression in hippocampus alleviated autophagy impairment and AD related neuropathological changes in 3xTg AD mice. (**A-G**) 2 months after control AAV or CCZ1-MON1A AAV injection, the hippocampus tissues of mice were dissected and subjected to immunoblotting analysis of LC3-II, SQSTM1, FL-APP and APP-CTFs. Data are quantified as mean ± SEM (n = 9). *P < 0.05, **P < 0.01, *vs.* the relative control. (**H**) The mice brain sections were stained with 4G8 antibody to visualize the Aβ plaques and intraneuronal Aβ accumulation. Bar: 200 μm. (**I-J**) Intracellular Aβ1-40 and Aβ1-42 in mice hippocampus were measured by ELISA. Data are quantified as mean ± SEM (n = 9). *P < 0.05, **P < 0.01, *vs.* the relative control. (**K-N**) The phosphorylated tau in mice hippocampal lysates were determined by immunoblotting using antibodies indicated on the figure. Data are quantified as mean ± SEM (n = 9). *P < 0.05, **P < 0.01 *vs.* the relative control.

Next, we examined autophagy status. We found that over-expression of CCZ1-MON1A significantly increased the protein level of LC3-II and reduced SQSTM1 level in 3xTg AD mice and WT mice (**Figure 8A-C, Figure S7A-F**), suggesting that autophagy was enhanced in CCZ1-MON1A-injected 3xTg AD mice and WT mice. Furthermore, over-expression of CCZ1-MON1A decreased the levels of FL-APP, APP-CTFs (**Figure 8D-G**), and intracellular Aβ1-42/Aβ1-40, and of the Aβ plaque load significantly (**Figure 8H-J**). The levels of phosphorylated tau (S205/T202, S214, T181) also dramatically decreased in CCZ1-MON1A over-expression mice. (**Figure 8K-N**). Conversely, AAV-mediated *Mon1a* KD exacerbated autophagy impairment in 3xTg AD mice, and AD-related behavioral and neuropathological changes in 3xTg AD mice. AAV-GFP-sh-Mon1a injection aggravated the spatial memory impairment and anxiety as shown by the MWM, Y-maze and open field test results (**Figure S5E-N**). The injection also inhibited autophagy as evidenced by accumulation of LC3 and SQSTM1 (**Figure S6A-C Figure S7G-L**), resulting in significant accumulation of FL-APP, APP-CTFs, Aβ plaques, intracellular Aβ1-42/Aβ1-40 and P-tau (**Figure S6D-N**). Collectively, CCZ1-MON1A over-expression in the hippocampus improved cognitive and emotional performance, restored autophagy function, and alleviated neuropathology in a 3xTg AD mouse model, indicating that CCZ1-MON1A over-expression is neuroprotective in this model.

## Material and Methods

### Reagents

Antibodies for Immunoblotting and IP, anti-ACTB/β-actin (sc-47778), and anti-CCZ1 antibody (sc-514290), were purchased from Santa Cruz Biotechnology. Anti-flag antibody (14794), anti-GFP (2956), anti-GST antibody (2622), anti-RAB7 antibody (9467), ATG5 antibody (9980), ATG7 antibody (8558), anti-rabbit IgG (7074), anti-mouse IgG (7076) and normal rabbit IgG (2729) were purchased from Cell Signaling Technology. Anti-LC3B (NB100-2220) antibody and anti-MON1A (NBP1-52007) were purchased from Novus Biologicals. Anti-hVPS34 antibody (Z-R016 and Z-R015) was purchased from Echelon Bioscience. Anti-SQSTM1/p62 (P0067) was purchased from Sigma-Aldrich. For immunofluorescence studies, Alexa Fluor® 488 goat anti-rabbit/mouse IgG (A-11008, A-11001), Alexa Fluor® 555 goat anti-rabbit/mouse IgG (A-21429, A-21424) and Alexa Fluor® 647 goat anti-rabbit/mouse IgG (A-21244, A-21235) were purchased from Thermo Fisher Scientific. For ELISA, anti-Aβ1-16 monoclonal antibody (6E10, 803001) and anti-beta amyloid polyclonal antibody (CT695, 51-2700) were purchased from Thermo Fisher. Anti-beta-amyloid 1-42 antibody (AB5078P) was purchased from Millipor. Anti-beta-amyloid 1-40 antibody (0060-100/bA4(40)-5C3) was purchased from Nanotools. DMEM (11965-126). Dynabeads® protein G for immunoprecipitation (10003D), fetal bovine serum (10270-106), opti-MEM (31985-070), G418 (10131-035), lipofectamine™ 3000 Transfection Reagent (L3000008), mant-GDP (M12414) and GTP (18332015) were purchased from Thermo Fisher Scientific.

### Cell culture and transfection

N2S (N2a stably transfected with human Swedish mutant cell) cells were cultured in Dulbecco's Modified Eagle's Medium (DMEM) containing 10% fetal bovine serum and 200 ug/ml G418, in a humidified atmosphere of 5% CO_2_ at 37 °C. Hela-GFP-LC3 cells and N2a cells were cultured in DMEM containing 10% fetal bovine serum in a humidified atmosphere of 5% CO_2_ at 37 °C. Cells were transfected with plasmids using Lipofectamine® 3000 Transfection Reagent (L300008) from Thermo Fisher according to the manufacturer's instructions. 24-72 h after transfection, cells were analyzed.

### Immunostaining

Cell lines were trypsinized and transferred to 24-well dishes containing coverslips followed by transient transfection with indicated plasmids for 24-72 h. Then cells were fixed using ice-cold formaldehyde for 10 min. Cell were then permeabilized in 0.2% Triton X-100 (Sigma, T8787). Cells were incubated with primary antibodies at 4 °C overnight, washed with PBS buffer and then incubated with secondary antibodies for 2 h at room temperature. Cells were then washed 3 times with PBS and stained with DAPI at room temperature for 5 min. Slides were examined with a confocal microscope.

### Immunoblotting

Cells or tissues were homogenized in ice-cold RIPA buffer (Cell Signaling Technology, 9806) with complete protease inhibitor mixture (Roche Applied Science, 04693124001). The proteins were separated by 10-15% SDS-polyacrylamide gel electrophoresis and transferred to PVDF membranes. The membranes were blocked with 5% nonfat milk dissolved in TBST for 2 h and incubated with primary antibodies overnight at 4 °C. Finally, the blots were incubated with secondary antibody 2 h at room temperature. Blots were visualized using ECL kit (GE Healthcare Life Sciences, RPN2235).

### MON1, RAB7 mRNA analysis

Gene expression was examined at the mRNA level in multiple brain regions of healthy control and AD patient samples from the Mount Sinai Brain Bank (https://www.synapse.org/#!Synapse:syn3157743). Spearman correlation analysis was performed to examine the relationship between gene expression and CDR score with Benjamini-Hochberg correction for multiple testing.

### Purification of autophagosomes from cells and mouse brains

Cells or brain tissue was homogenized in homogenization buffer (0.25 M sucrose, 10 mM HEPES, 1 mM EDTA, protease inhibitors pH 7.5), then centrifuged at 1000 g for 5 min. Lysosomes and nuclei were removed by glycyl-phenylalanine-naphthylamide (GPN). We removed mitochondria using different concentrations of Nycodenz. The final autophagosome proteins were extracted using ice-cold RIPA buffer (Cell Signaling Technology, 9806) 32.

### GTP-agarose pull-down for RAB7 activity assay

GTP-RAB7 was measured by GTP-agarose beads (Sigma, G9768) following a published protocol 24. Briefly, cells or animal brains were collected and lysed in a buffer containing 50 mM Tris-HCl pH 7.5, 5 mM MgCl_2_, 0.5% Triton X-100, 250 mM NaCl, and protease inhibitors. The solution was then incubated with GTP-beads overnight at 4 °C. The beads were washed with lysis buffer and boiled in SDS-loading buffer before SDS-PAGE for immunoblotting.

### GST-R7BD pull-down assay

GTP-Rab7 was purified using GST-R7BD bait. Mammalian cells were lysed by the lysis (pull down) buffer (100 mM NaCl, 20 mM HEPES, 1% Triton X-100, 5 mM MgCl_2_, protease inhibitors). Each pull-down was performed in 1 mL with 500 µg of cell lysate and 100 µL of beads pre-equilibrated in pull-down buffer. Beads were rotated overnight at 4 °C, and washed twice with cold pull-down buffer. Bound proteins were eluted by adding 1× sample buffer, and then incubated at 95 °C for 8 min. The amount of GTP- RAB7 was measured by blotting with a RAB7-specific antibody and appropriate secondary antibody.

### GEF assay

GEF assays were performed as follows. Mant-GDP-bound RAB7 was diluted to a concentration of 50 nM in exchange buffer [20 mM HEPES (pH 7.5), 0.5 mM MgCl_2_ and 150 mM NaCl 15. The dissociation of mant-GDP from RAB7 was determined by measuring the decrease in fluorescence signal that accompanies release of mant-GDP in the presence of a large excess of GTP. Nucleotide release reactions were initiated by addition of GTP (1 mM final concentration) to the reaction buffer. The reaction buffer was then incubated with purified CCZ1-MON1A from cell or mouse brain. Samples were excited at 360 nm, and the emission was monitored at 440 nm.

### PIK3C3/VPS34 kinase assay

PIK3C3/VPS34 kinase activity was determined by Class Ⅲ PI3K ELISA kit (Echelon, cat: K-3000). Briefly, VPS34 protein was immunoprecipitated by VPS34 antibody or CCZ1 antibody. Prepare PI for kinase reaction. The kinase reaction was allowed to proceed for 2 h at 37 °C. The kinase reaction was quenched by adding EDTA. This solution was then incubated with PI3P detector at room temperature for 1 h. The solution was discarded, each plate was washed 3 times with TBST. Secondary detector was added, and solutions were incubated for 30 min at room temperature. The solution was again discarded, and each plate was washed 3 times with TBST. TMB solution was added, and incubated for 30 min at room temperature. H_2_SO_4_ was added to stop the reaction. Absorbance values were read at 450 nm.

### ELISA assay of Aβ1-40 and Aβ1-42

The brain tissues and cells were lysed in lysis buffer (Cell Signaling Technology, 9806). ELISA plates (Nunc) were coated with 6E10 antibody, and incubated at 4 °C overnight. Plates were washed 3 times with PBST and blocked with Block Ace for 2 h at room temperature. Plates were again washed with PBST 3 times. Samples or standards were incubated at 37 °C for 2 h. Plates were washed with PBST 3 times. Secondary antibody was added, and incubated for 2 h at 37 °C. Plates were washed 4 times with PBST. Streptavidin-conjugated horseradish peroxidase was prepared in a ratio of 1:4000 in assay diluent, and dispensed 100 μl/well. Wells were incubated for 1 h at 37 °C. The wells were washed 4 times with PBST. A solution of Buffers A and B in 1:1 ratio was prepared and added, 100 μl/well; wells were incubated for 30 min in dark. H_2_SO_4_ was added to stop the reaction. Absorbance at 450 nm was read and recorded.

### Mice feeding

3xTg AD mice and C57 mice were housed in individually ventilated cages with standard rodent bedding. The room temperature was kept at approximately 22 °C and the relative humidity between 40 and 70%. Mice were housed under constant light-cycle (12 h light/dark). All experiments were carried out in accordance with the recommendations in the Guide for the Care and Use of Laboratory Animals of the National Institutes of Health. The protocol was approved by the University of Macau research ethics committee (Approval No. UMARE-013-2019).

### Stereotactic injection of adeno-associated virus

Adeno-associated virus (AAV) was stereotactically, injected bilaterally into 3xTg AD mouse hippocampi as described. AAV-Flag-*ccz1*, AAV-Flag-*mon1a*, AAV-Flag, AAV-GFP-sh-*mon1a* and AAV-GFP-sh-Ctrl were stereotactically injected into the hippocampi of 6-month-old 3xTg AD mice at the following coordinates: anterior posterior, 2.0 mm; medial lateral, ± 1.7 mm; dorsal ventral, 2.0 mm.

### Morris water maze

The Morris water maze was used to evaluate spatial learning memory. The experiments were performed in a 100 cm diameter, 50 cm deep tank filled with opaque water kept at 21 °C. The 10 cm diameter platform was submerged 1 cm under the water surface. Training trials lasted 60 sec, and were performed 4 times a day on 5 consecutive days. The platform location was held constant within each pair of daily tests, but the location of the animal's starting point was changed for each test. To assess long-term spatial memory retrieval, a probe trial (60 s) was conducted 24 h a after the last training session. The time spent in the target quadrant was recorded.

### Y-maze test

Mice short-term memory was assessed using the Y maze. There are 3 arms in a Y maze: novel arm, starting arm, and other arm. During the first stage, the novel arm is blocked. Mice entering the starting arm are allowed to move into the open arm for 5 min. After 2 h, the novel arm is opened, starting the second phase. The time spent in each arm, and the number of entries into each arm is recorded.

### Open field test

Anxiety-related behavior of the mice was measured by the open field test. A 60 cm× 60 cm× 25 cm box, with a 30 cm× 30 cm outlined center area, was constructed. Each mouse was placed in the center area and left for 5 min. The time spent in the center area and at the margins was recorded.

### Statistical analysis

Each experiment was performed at least 3 times, and the results are presented as mean ± SEM. One-way analysis of variance (ANOVA) was followed by the Student-Newman-Keuls test using the Sigma Plot 11.0 software package. A probability value of P< 0.05 was considered to be statistically significant.

## Discussion

Accumulating evidence implicates autophagic-lysosomal pathway dysregulation in AD 33. Consistent with this evidence, our findings reported here showed that LC3-II and p62 accumulated in multiple cellular and animal models of AD (Figure 1). Furthermore, our data reveals, for the first time, that the CCZ1-MON1A-RAB7 module is impaired in AD models, suggesting a novel pathogenic mechanism for autophagosome maturation inhibition. We showed that over-expression of CCZ1-MON1A enhanced autophagosome maturation, promoted APP-CTFs, Aβ and P-tau degradation, and alleviated memory defects and neuropathology in AD models. This study highlights the critical role of the CCZ1-MON1A-RAB7 module in the regulation of neuronal autophagy to maintain neuron homeostasis and counteract neurodegeneration.

The CCZ1-MON1A complex is a RAB7 GEF which promotes the transformation of GDP-RAB7 (inactive state) into GTP-RAB7 (active state). RAB7 plays a key role in endosome and autophagosome maturation by promoting vesicle transportation 15. RAB7 is required for the transfer of the cargo from late endosomes to lysosomes, and RAB7 is activated by the CCZ1-MON1A complex on late endosomes and it dissociates from lysosomes in mammalian cells 34. CCZ1 directly interacts with Atg8 to become localized on autophagosomes 18. However, the role of CCZ1-MON1A in the regulation of autophagy in mammals is still unclear. Here, our results show that, in neuronal cells and mouse brains, CCZ1-MON1A over-expression increased the active form of RAB7 and promoted autophagosome maturation, while *Mon1a* depletion blocked autophagosome maturation. Our previous study found that APP interacts with CCZ1, and the interaction may contribute to the redistribution of APP and APP-CTF in neuronal cells. Indeed, over-expression of CCZ1-MON1A increased the recruitment of APP/APP-CTFs to autophagosomes and lysosomes, and this phenomenon was more obvious after CQ treatment. These data indicate that CCZ1-MON1A, by generating an active form of RAB7, plays a key role in regulating autophagosome maturation. Interestingly, over-expression of CCZ1-MON1A attenuated Aβ and tau pathology in cellular and mice models of AD, and alleviated memory impairment in 3xTg AD mice. The results suggest that CCZ1-MON1A is a critical factor in the regulation of Aβ and tau pathology, as well as in memory in AD. However, further study is needed to clarify whether CCZ1-MON1A exerts anti-AD effects mainly through autophagy or whether other mechanisms are involved.

Abnormal accumulation of autophagosomes can be observed in AD animal models and AD patients, implying the interruption of autophagosome maturation 5. Several mechanisms have been found to be responsible for the impaired autophagosome maturation, including lysosomal inhibition. Though enhancement of autophagy has been regarded as a therapeutic strategy against AD, simply activating autophagosome biogenesis may be problematic as it will lead to autophagosome accumulation when maturation is inhibited. How to selectively restore impaired autophagosome maturation is still an unsolved problem. By revealing the impaired CCZ1-MON1A GEF activity in AD models and showing that over-expression of CCZ1-MON1A promoted autophagosome maturation and alleviated autophagic substrates accumulation in cellular and mice models of AD, we show that modulation of CCZ1-MON1A expression or CCZ1-MON1A GEF activity can be an approach to restoring autophagosome maturation in AD. A further task would be to identify small molecule CCZ1-MON1A activator(s) for therapeutic applications.

In summary, our study revealed the dysregulation of CCZ1-MON1A-RAB7 in AD models, and showed that CCZ1-MON1A is the positive regulator of autophagosome maturation for efficient clearance of APP-CTF, Aβ and P-tau in AD models. Our work thus establishes an important link between CCZ1-MON1A dysfunction, autophagosome maturation impairment and AD development. More broadly, our study results support the concept that enhancing autophagosome maturation might be a viable therapeutic strategy against AD.

## Supplementary Material

Supplementary figures.

## Abbreviations

3xTg AD: triple transgenic mouse for Alzheimer's disease
Aβ: amyloid beta peptide
Aβ1-40: amyloid beta peptide 1-40
Aβ1-42: amyloid beta peptide 1-42
AD: Alzheimer's disease
APP: amyloid beta precursor protein
APP-CTFs: APP C-terminal fragments
ATG: autophagy related
ATG5: autophagy related 5
ATG7: autophagy related 7
CCZ1: CCZ1 homolog, vacuolar protein trafficking and biogenesis associated
CQ: chloroquine
DAPI: 4',6-diamidino-2-phenylindole
GDP: guanine diphosphate
GEF: guanine nucleotide exchange factor
GTP: guanine triphosphate
GTPase: guanosine triphosphatase
KD: knockdown
KO: knockout
LAMP1: lysosomal associated membrane protein 1
MAP1LC3/LC3: microtubule associated protein 1 light chain 3
MON1A: MON1A homolog, secretory trafficking associated
MWM: Morris water maze
NRBF2: nuclear receptor binding factor 2
PtdIns3P: phosphatidylinositol-3-phosphate
RILP: RAB interacting lysosomal protein
SQSTM1/p62: sequestosome 1
VPS: vacuolar protein sorting
WT: wild type
