# Supplementary material for: Enhancing autophagy maturation with CCZ1-MON1A complex alleviates neuropathology and memory defects in Alzheimer disease models
Source: Theranostics. 2022 Jan 24;12(4):1738–55. doi: 10.7150/thno.64148 (PMC8825577; doi:10.7150/thno.64148)
Supplement: Supplementary file 1 — Supplementary figures. [file thnov12p1738s1.pdf]

Supplementary material

Enhancing autophagy maturation with CCZ1-MON1A complex alleviates neuropathology and memory defects in Alzheimer disease models

Cui-Zan Cai,[1,2] Xu-Xu Zhuang,[1] Qi Zhu,[1] Ming-Yue Wu,[1] Huanxing Su,[1] Xiao-jin Wang,[2] Ashok Iyaswamy,[3] Zhenyu Yue,[4] Qian Wang,[4,5] Bin Zhang,[4,5,6,7] Yu Xue,[8] Jieqiong Tan,[9] Min Li,[3] Huanhuan He,[2,] * and Jia-Hong Lu[1],*

[1]State Key Laboratory of Quality Research in Chinese Medicine, Institute of Chinese Medical Sciences, University of Macau, Macau SAR, China

[2]Molecular Imaging Center, Guangdong Provincial Key Laboratory of Biomedical Imaging, The Fifth Affiliated Hospital, Sun Yat-sen University, Zhuhai, 519000, China

[3]Mr. and Mrs. Ko Chi Ming Centre for Parkinson's Disease Research, School of Chinese Medicine, Hong Kong Baptist University, Hong Kong SAR, China

[4]Department of Neurology, The Friedman Brain Institute, Icahn School of Medicine at Mount Sinai, New York, NY, 10029, USA

[5]Mount Sinai Center for Transformative Disease Modeling, Icahn School of Medicine at Mount Sinai, New York, NY 10029, USA

[6]Department of Pharmacological Sciences, Icahn School of Medicine at Mount Sinai, New York, NY 10029, USA

[7]Icahn Genomics Institute, Icahn School of Medicine at Mount Sinai, New York, NY 10029, USA

[8]Key Laboratory of Molecular Biophysics of Ministry of Education, Hubei Bioinformatics and Molecular Imaging Key Laboratory, Center for Artificial Intelligence Biology, College of Life

Science and Technology, Huazhong University of Science and Technology, Wuhan, China

[9]Center for Medical Genetics, School of Life Sciences, Central South University, Changsha 410078, China

* Authors for correspondence: hehh23@mail.sysu.edu.cn (Huanhuan He); jiahonglu@um.edu.mo (Jia-Hong Lu)

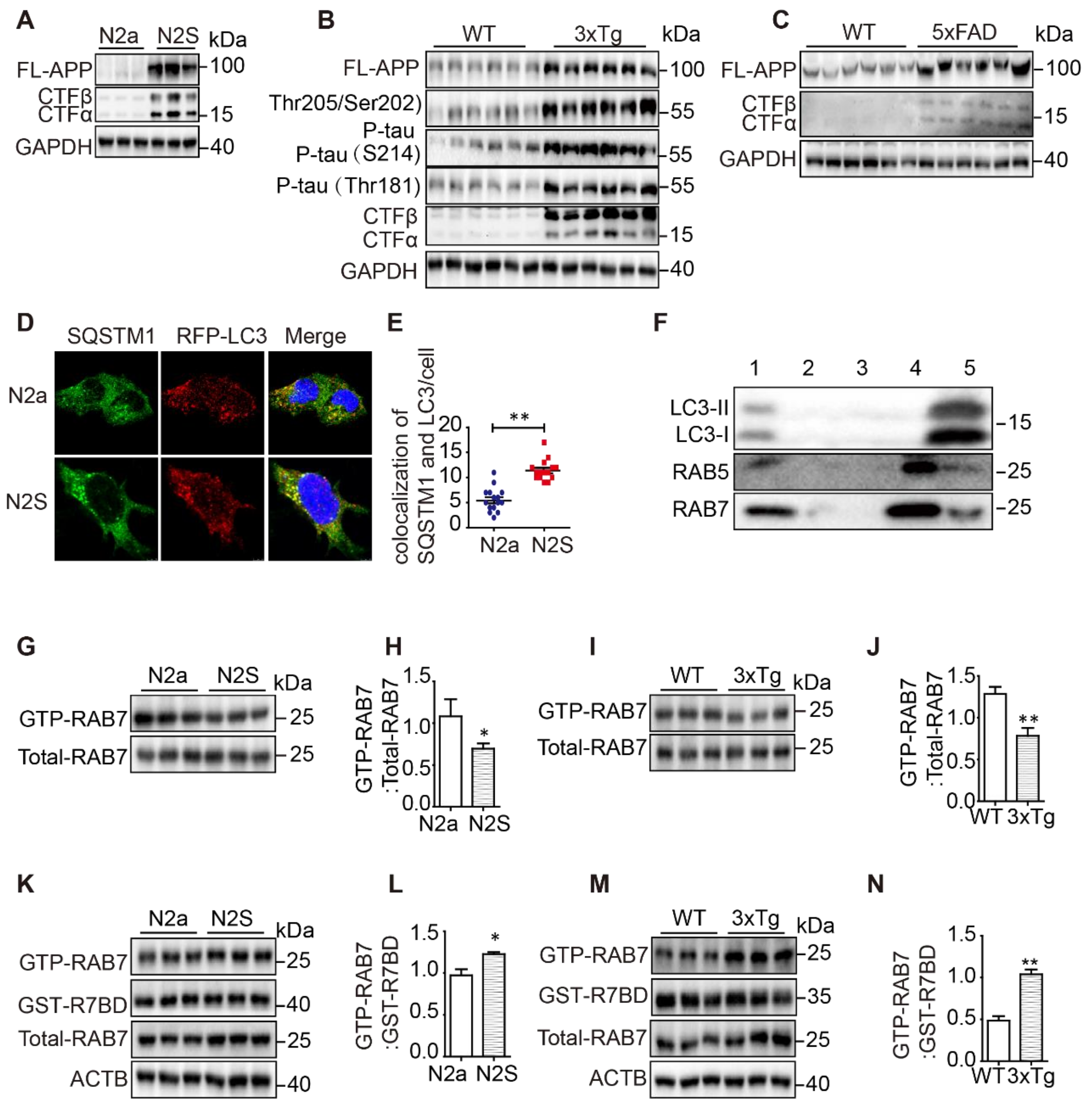

Figure S1. (**A**) Immunoblotting was used to detect the expression of FL-APP, and APP-CTFs levels in N2a and N2S, GAPDH was used as an internal control. (**B**) Immunoblotting was used to detect the expression of FL-APP, and APP-CTFs levels in the hippocampus region of 12-month-old C57BL/6 and 3xTg AD mice; GAPDH was used as an internal control. (**C**) Immunoblotting was used to detect the expression of FL-APP, and APP-CTFs levels in the hippocampus region of 12-month-old WT and 5xFAD AD mice; GAPDH was used as an internal control. (**D-E**) N2a and N2S cells were transiently transfected with RFP-LC3, and the

colocalization of RFP-LC3 with endogenous SQSTM1 was visualized under confocal microscopy. (**F**) Enrichment of LC3-II in purified autophagosomes. (**G-H**) GTP-RAB7 in autophagosomes isolation from N2a and N2S were determined by GTP-beads affinity-isolation assay. Data are quantified as mean ± SEM (n = 3). *P < 0.05, **P < 0.01, *vs*. the relative control. (**I-J**) GTP-RAB7 in autophagosomes isolation from WT and 3xTg were determined by GTP-beads affinity-isolation assay. Data are quantified as mean ± SEM (n = 3). *P < 0.05, **P < 0.01, vs. the relative control. (**K-L**) The total amount of GTP-RAB7 in whole cell from N2a and N2S were determined by GST-R7BD affinity-isolation assay. Data are quantified as mean ± SEM (n = 3). *P < 0.05, **P < 0.01, *vs*. the relative control. (**M-N**) The total amount of GTP-RAB7 in whole cell from WT and 3xTg AD mouse were determined by GST-R7BD affinity-isolation assay. Data are quantified as mean ± SEM (n = 3). *P < 0.05, **P < 0.01, *vs*. the relative control.

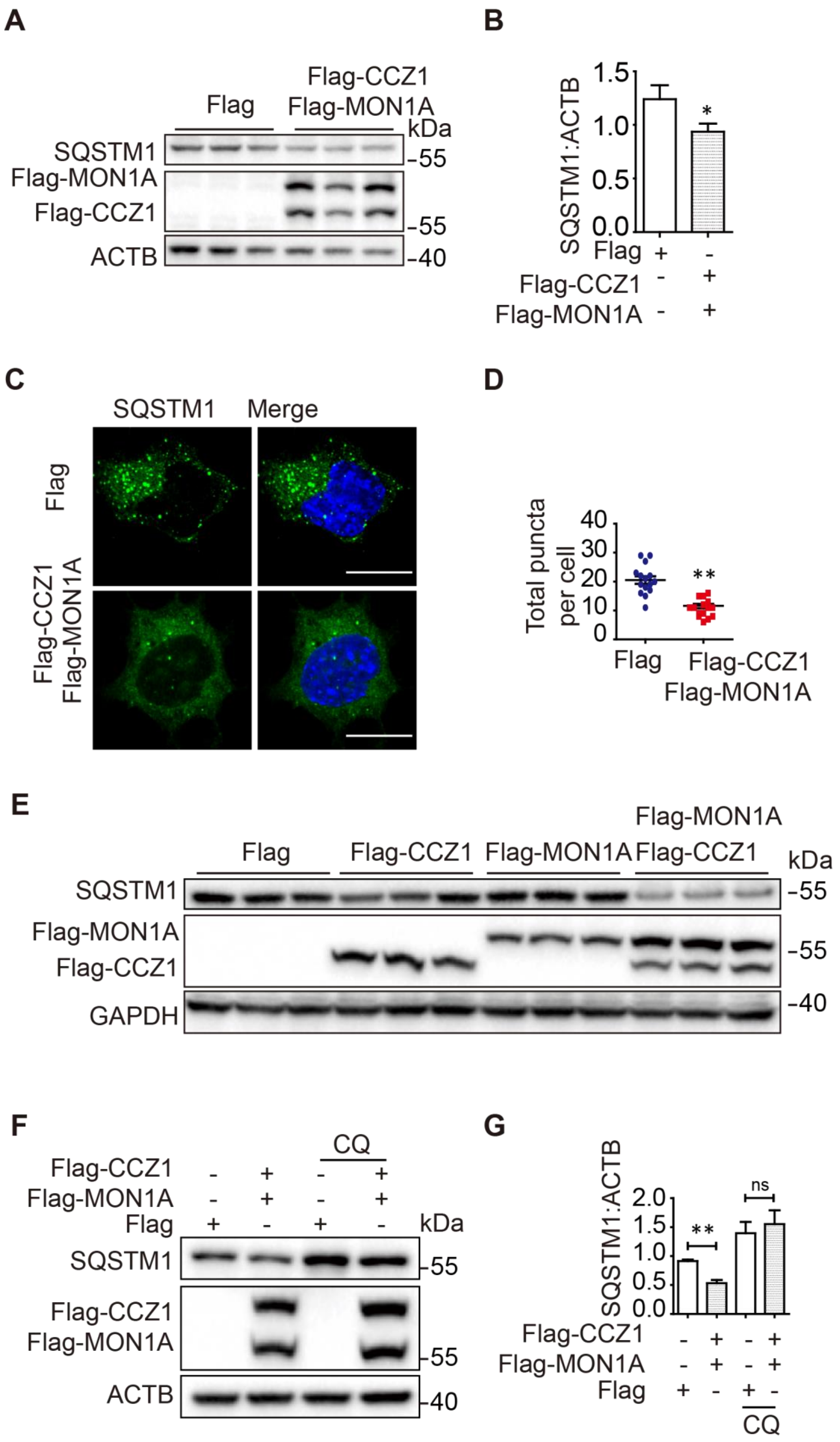

A
Flag
Flag-CCZ1
Flag-MON1A
kDa
SQSTM1
Flag-MON1A
Flag-CCZ1
ACTB
55
55
40
B
SQSTM1:ACTB
1.5
1.0
0.5
0.0
*
Flag + -
Flag-CCZ1 - +
Flag-MON1A - +
C
SQSTM1
Merge
Flag
Flag-CCZ1
Flag-MON1A
D
Total puncta per cell
40
30
20
10
0
**
Flag
Flag-CCZ1
Flag-MON1A
E
Flag
Flag-CCZ1
Flag-MON1A
Flag-MON1A
Flag-CCZ1
kDa
SQSTM1
Flag-MON1A
Flag-CCZ1
GAPDH
55
55
40
F
CQ
Flag-CCZ1 - + - +
Flag-MON1A - + - +
Flag + - + -
kDa
SQSTM1
Flag-CCZ1
Flag-MON1A
ACTB
55
55
40
G
SQSTM1:ACTB
2.0
1.5
1.0
0.5
0.0
**
ns
Flag-CCZ1 - + - +
Flag-MON1A - + - +
Flag + - + -
CQ

Figure S2. (**A-B**) SQSTM1 levels were determined by immunoblotting after transfection of FLAG-CCZ1/FLAG-MON1A or FLAG plasmid in N2S cells. (**C-D**) SQSTM1 levels were determined by immunofluorescence after transfection of FLAG-CCZ1/FLAG-MON1A or FLAG plasmid in N2S cells. Quantification data were presented as the mean ± SEM, n = 20-25 cells from 3 independent experiments. *P < 0.05, **P < 0.01, *vs*. the relative control. Scale bar: 5 μm. (**E**) SQSTM1 levels were determined by immunoblotting after transfection of FLAG-CCZ1 and FLAG-MON1A plasmids alone or in combination. (**F-G**) N2a cells were transfected with FLAG-CCZ1/FLAG-MON1A plasmids and co-treated with vehicle or 30 μM of CQ for 12 h. Immunoblotting was used to detect the SQSTM1 levels. *P < 0.05, **P < 0.01, ***P < 0.001 *vs*. the relative control.

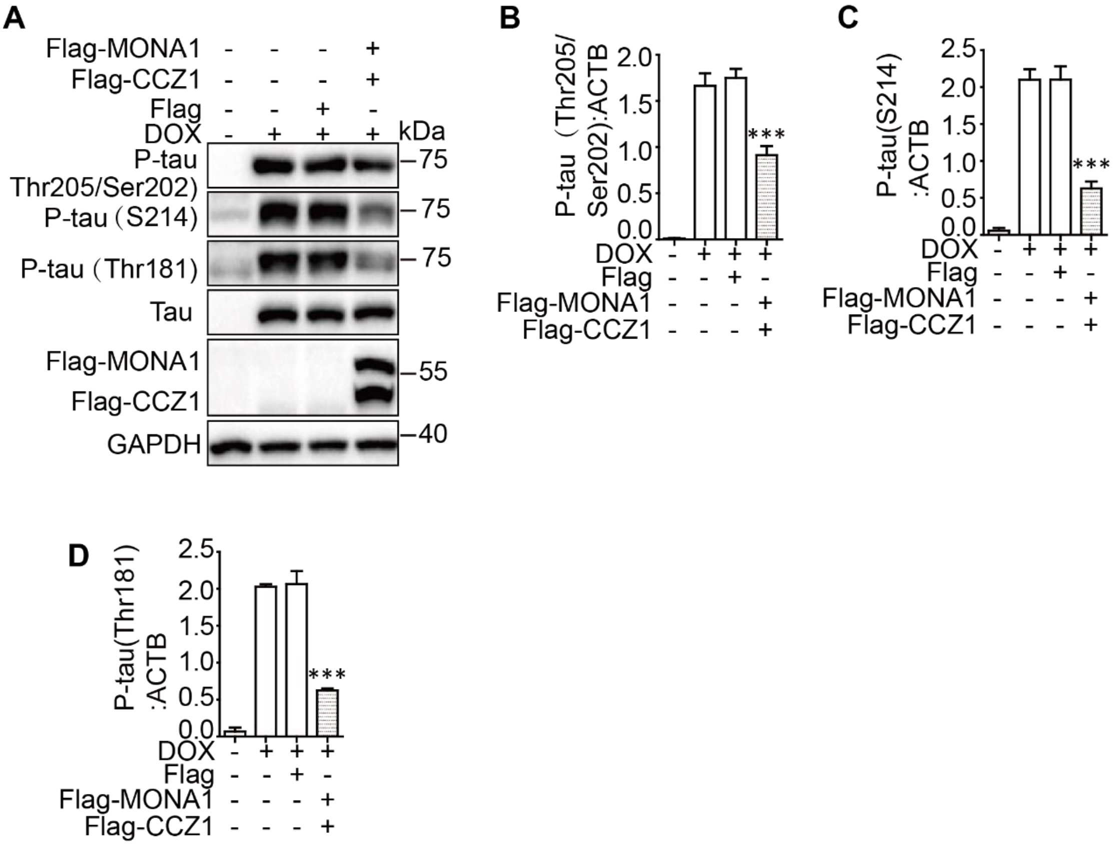

Figure S3. (**A-D**) HEK 293 cells expressing pTRE3G-mcherry-BI promoter-EGFP Tau P301L (HEK 293 3G-EGFP-Tau P301L/mCherry) were treated with doxycycline (DOX) for 24 h to induce the expression of Tau P301L. Then the cell was transfected with Flag-CCZ1, Flag-MON1A and Flag plasmids, and the levels of P-tau, Flag-CCZ1 and Flag-MON1A were examined by immunoblotting. Data are quantified as mean ± SEM (n = 3). *$P < 0.05$, **$P < 0.01$, *vs*. the relative control.

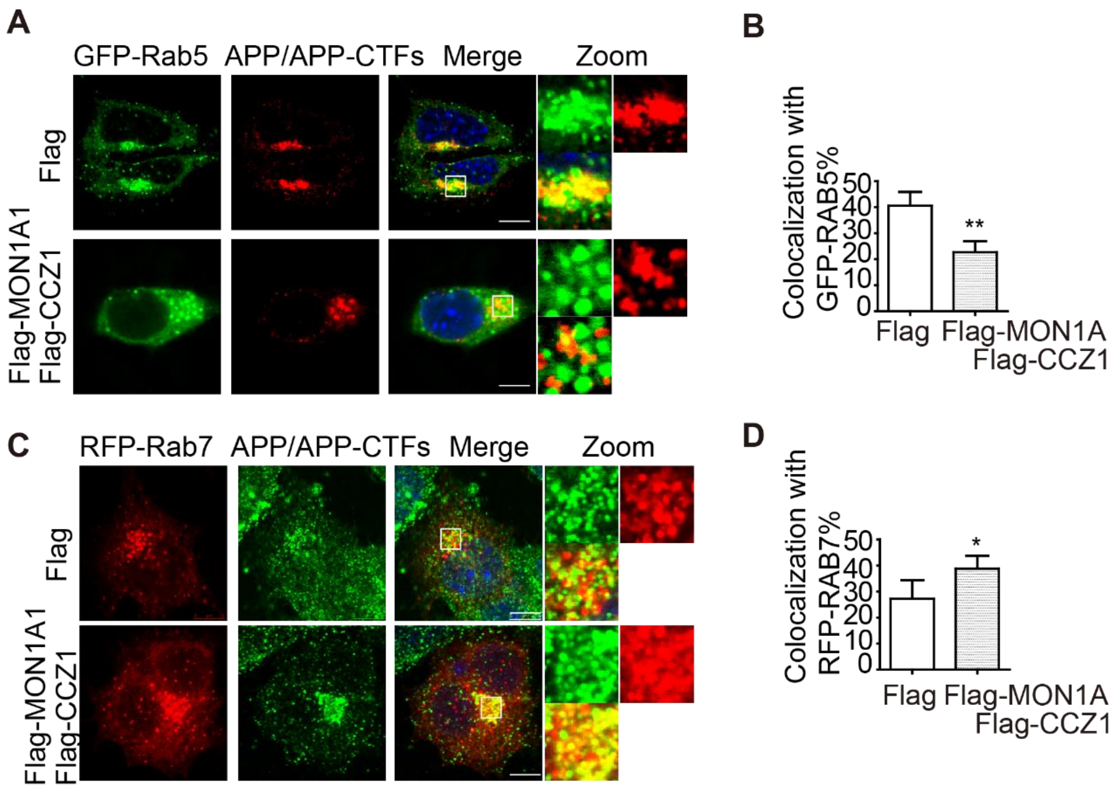

Figure S4. (**A-B**) N2S cells were transiently transfected with GFP-RAB5 and stained with APP antibody. The colocalization of GFP-RAB5 and APP/APP-CTFs was visualized under confocal microscope. Quantification data were presented as the mean ± SEM, n = 20-25 cells from 3 independent experiments. Scale bar: 7.5 μm. $^*P < 0.05$, $^{**}P < 0.01$, *vs*. the relative control. (**C-D**) N2S cells were transiently transfected with RFP-RAB7 and stained with APP antibody. The colocalization of RFP-RAB7 and APP, APP-CTFs was visualized under confocal microscope. Quantification data were presented as the mean ± SEM, n = 20-25 cells from 3 independent experiments. $^*P < 0.05$, $^{**}P < 0.01$, *vs*. the relative control. Scale bar: 7.5 μm.

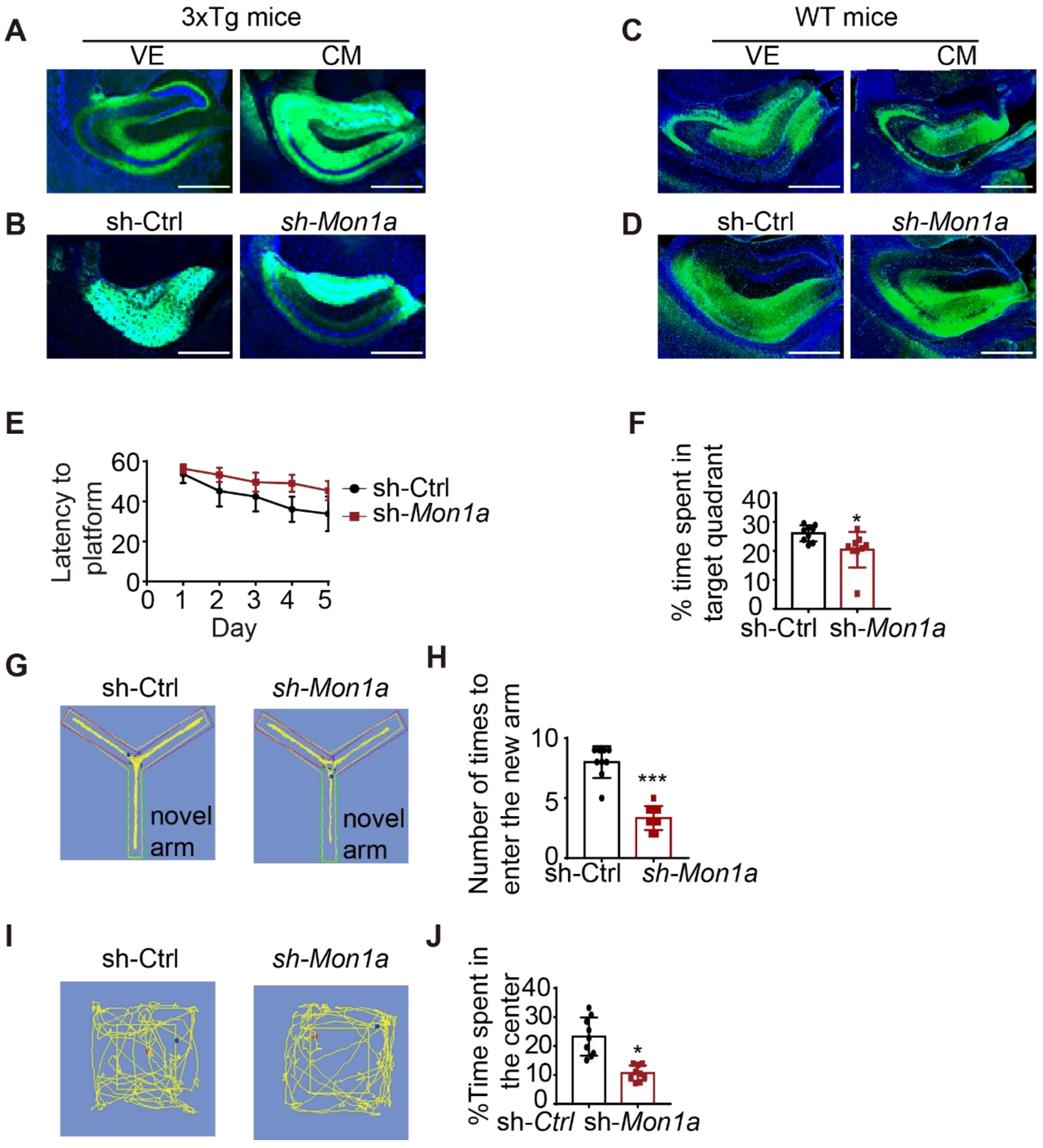

Figure S5. (**A**) Representative image of AAV-Flag-ccz1and AAV-Flag-mon1ainjection of hippocampus. AAV-Flag-Ccz1, AAV-Flag-Mon1 efficiently transduces all types of neurons of the hippocampus in 3xTg AD mouse. bar: 800 μm. (**B**) Representative image of AAV-GFP-sh-mon1ainjection of hippocampus. AAV-GFP-sh-mon1a, AAV-GFP-sh-Ctrl efficiently transduces all types of neurons of the hippocampus in 3xTg AD mouse. bar: 8000 μm. (**C**) Representative image of AAV-Flag-ccz1and AAV-Flag-mon1ainjection of hippocampus. AAV-Flag-Ccz1, AAV-Flag-Mon1 efficiently transduces all types of neurons of the

hippocampus in 12-month-old C57BL/6 WT mouse. bar: 800 μm. (**D**) Representative image of AAV-GFP-sh-mon1ainjection of hippocampus. AAV-GFP-sh-mon1a, AAV-GFP-sh-Ctrl efficiently transduces all types of neurons of the hippocampus in 12-month-old C57BL/6 WT mouse. bar: 8000 μm. (**E-F**) The spatial memory of 3xTg or 3xTg KD MON1A mice receiving AAV injection was evaluated by morris water maze (MWM). Training trials (60 sec each) were performed 4 times a day for 5 days and the time for mice to find the platform was recorded. After 5 days training, the platform was removed and the time that mice stay in the platform quadrant was recorded. Data are quantified as mean ± SEM (n = 9). *P < 0.05, **P <0.01, *vs*. the relative control. (**G-H**) Short-term memory was test by Y-maze. Data are quantified as mean ± SEM (n = 9). *P < 0.05, **P <0.01, *vs*. the relative control. (**I-J**) Open field test, representative exploratory patterns of mice in each group. Quantification of time spent in the center for each treatment. Data are quantified as mean ± SEM (n = 9). *P < 0.05, **P <0.01, *vs*. the relative control.

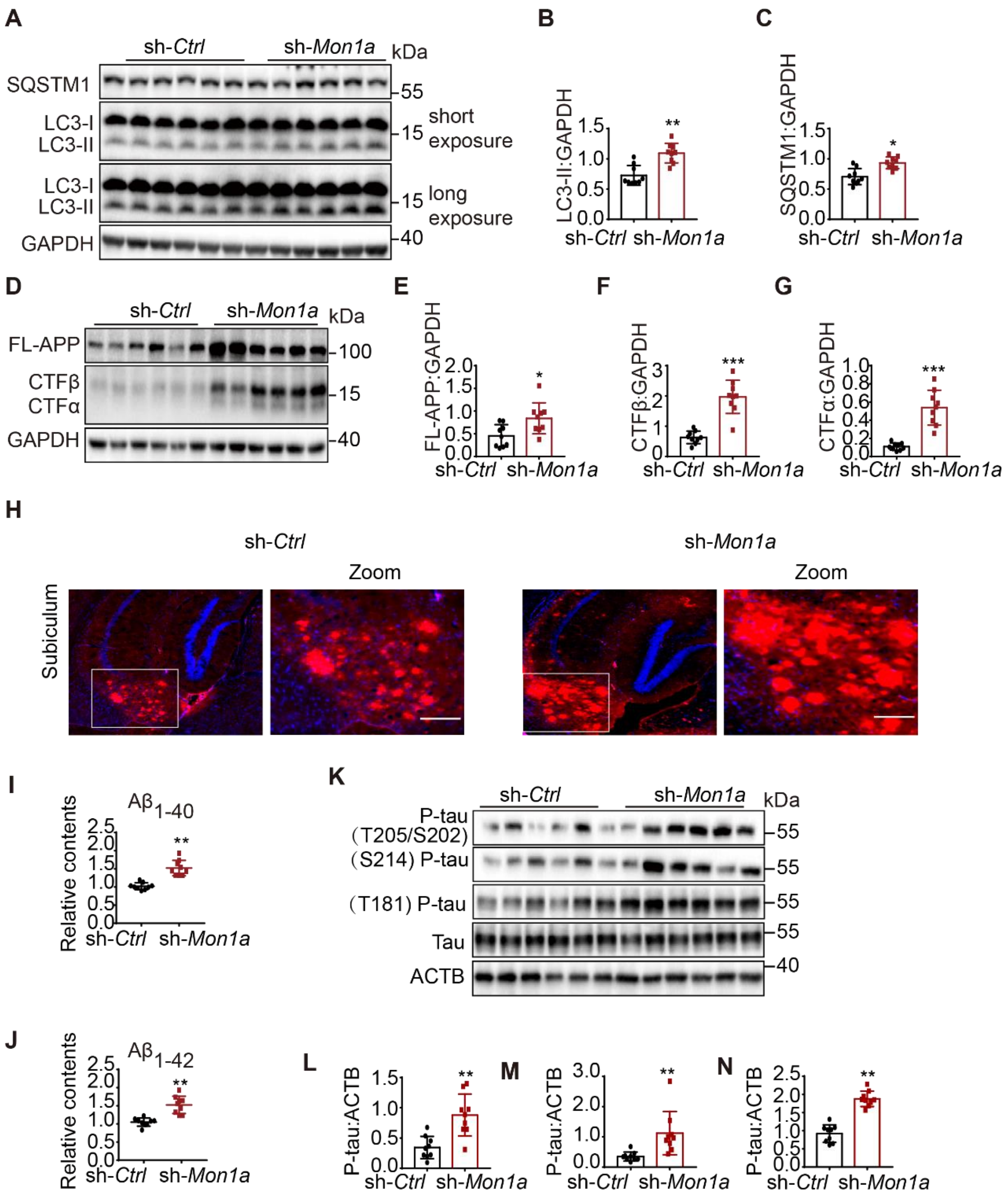

Figure S6. AAV-mediated CCZ1-MON1A knock down in hippocampus exacerbated autophagy impairment, APP/CTFs accumulation and Aβ deposits in 3xTg AD mice. (**A-G**) 2 months after control AAV or AAV-sh*Mon1a* injection, the hippocampus tissues of mice were dissected and subjected to immunoblotting analysis of LC3-II, SQSTM1, FL-APP and APP-CTFs. Data are quantified as mean ± SEM (n = 9). *P < 0.05, **P <0.01, *vs*. the relative control. (**H**) Representative images show the Aβ labeled with biotinylated 4G8 antibody. Data are

quantified as mean ± SEM (n = 9). *P < 0.05, **P <0.01, *vs*. the relative control. bar: 200 µm. (**I-J**) Intracellular Aβ1-40 and Aβ1-42 in mice hippocampus were measured by ELISA. Data are quantified as mean ± SEM (n = 9). *P < 0.05, **P <0.01, *vs*. the relative control. (**K-N**) The phosphorylated tau in mice hippocampal lysates were determined by immunoblotting using antibodies indicated on the figure. Data are quantified as mean ± SEM (n = 9). *P < 0.05, **P <0.01 *vs*. the relative control.

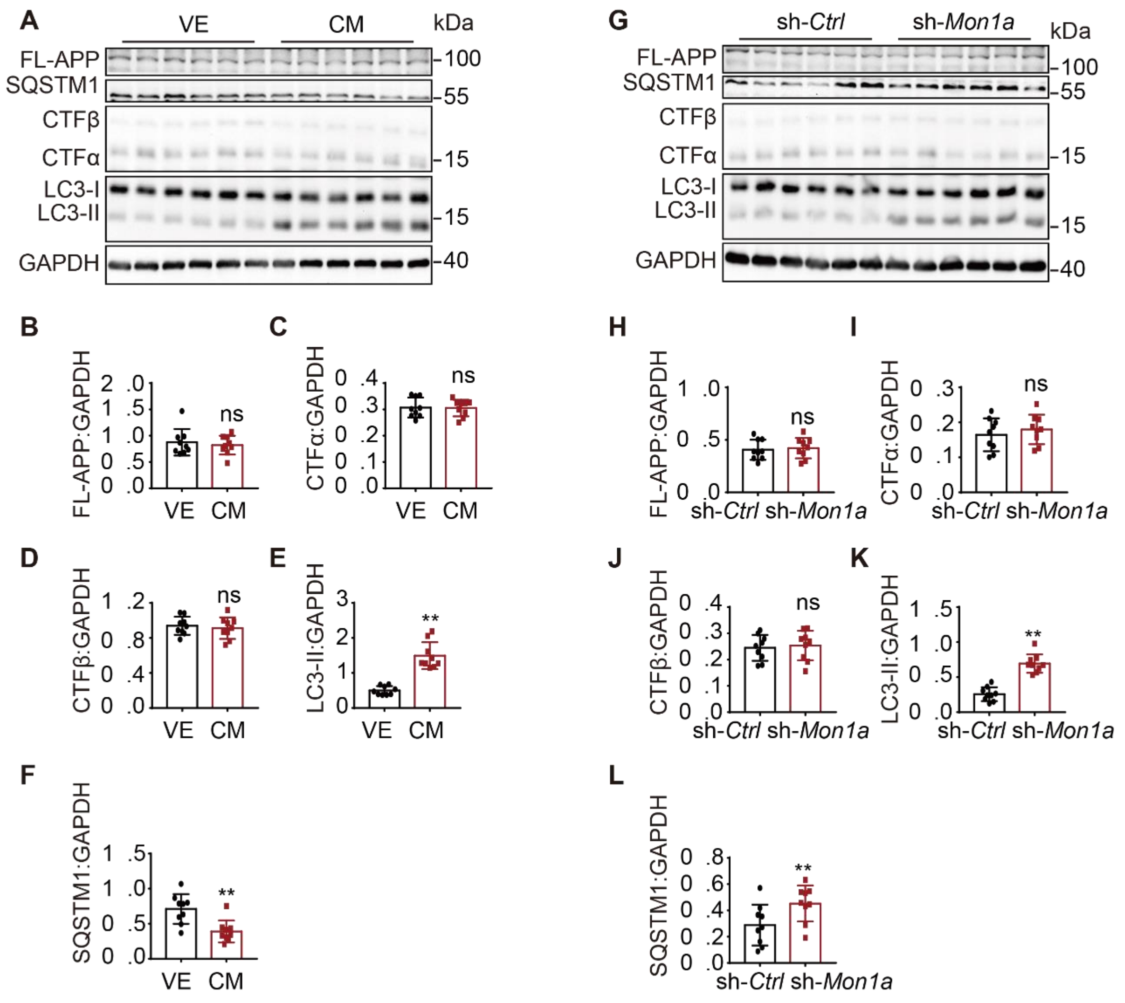

Figure S7. (A-F) 2 months after control AAV or CCZ1-MON1A AAV injection, the hippocampus tissues of mice were dissected and subjected to immunoblotting analysis of LC3-

II, SQSTM1, FL-APP and APP-CTFs. Data are quantified as mean ± SEM (n = 9). *P < 0.05, **P <0.01, *vs*. the relative control. ns, not significant. **(G-L)** 2 months after control AAV or AAV-sh*Mon1a* injection, the hippocampus tissues of mice were dissected and subjected to immunoblotting analysis of LC3-II, SQSTM1, FL-APP and APP-CTFs. Data are quantified as mean ± SEM (n = 9). *P < 0.05, **P <0.01, *vs.* the relative control.
